# Generative artificial intelligence and ethical considerations in health care: a scoping review and ethics checklist

**DOI:** 10.1016/S2589-7500(24)00143-2

**Published:** 2024-09-17

**Authors:** Yilin Ning, Salinelat Teixayavong, Yuqing Shang, Julian Savulescu, Vaishaanth Nagaraj, Di Miao, Mayli Mertens, Daniel Shu Wei Ting, Jasmine Chiat Ling Ong, Mingxuan Liu, Jiuwen Cao, Michael Dunn, Roger Vaughan, Marcus Eng Hock Ong, Joseph Jao-Yiu Sung, Eric J Topol, Nan Liu

**Affiliations:** Centre for Quantitative Medicine (Y Ning PhD, S Teixayavong BSS, Y Shang MSc, D Miao MSc, D S W Ting PhD, M Liu MSc, Prof R Vaughan PhD, N Liu PhD) and Programme in Health Services and Systems Research (Prof R Vaughan, Prof M E H Ong MPH, N Liu), Duke–NUS Medical School, Singapore; Centre for Biomedical Ethics, Yong Loo Lin School of Medicine, National University of Singapore, Singapore (Prof J Savulescu PhD, M Dunn PhD); Oxford Uehiro Centre for Practical Ethics, Faculty of Philosophy, University of Oxford, Oxford, UK (Prof J Savulescu); School of Medicine, Imperial College London, London, UK (V Nagaraj); Centre for Ethics, Department of Philosophy, University of Antwerp, Antwerp, Belgium (M Mertens PhD); Antwerp Center on Responsible AI, University of Antwerp, Antwerp, Belgium (M Mertens); Singapore Eye Research Institute, Singapore National Eye Centre, Singapore (D S W Ting); SingHealth AI Office, Singapore Health Services, Singapore (D S W Ting); Division of Pharmacy (J C L Ong PharmD) and Department of Emergency Medicine (Prof M E H Ong), Singapore General Hospital, Singapore; Machine Learning and I-Health International Cooperation Base of Zhejiang Province (Prof J Cao PhD) and Artificial Intelligence Institute (Prof J Cao), Hangzhou Dianzi University, Zhejiang, China; Lee Kong Chian School of Medicine, Nanyang Technological University, Singapore (Prof J J-Y Sung MD); Scripps Research Translational Institute, Scripps Research, La Jolla, CA, USA (Prof E J Topol MD); Institute of Data Science, National University of Singapore, Singapore (N Liu)

## Abstract

The widespread use of Chat Generative Pre-trained Transformer (known as ChatGPT) and other emerging technology that is powered by generative artificial intelligence (GenAI) has drawn attention to the potential ethical issues they can cause, especially in high-stakes applications such as health care, but ethical discussions have not yet been translated into operationalisable solutions. Furthermore, ongoing ethical discussions often neglect other types of GenAI that have been used to synthesise data (eg, images) for research and practical purposes, which resolve some ethical issues and expose others. We did a scoping review of the ethical discussions on GenAI in health care to comprehensively analyse gaps in the research. To reduce the gaps, we have developed a checklist for comprehensive assessment and evaluation of ethical discussions in GenAI research. The checklist can be integrated into peer review and publication systems to enhance GenAI research and might be useful for ethics-related disclosures for GenAI-powered products and health-care applications of such products and beyond.

## Introduction

In the past few years, development of Chat Generative Pre-trained Transformer (ChatGPT) and other chatbots powered by large language models (LLMs) have drawn the attention of the general public, researchers, and stakeholders to the fast developing technology of GenAI. GenAI differentiates from general AI technology due to its capacity to generate realistic content, such as text (eg, ChatGPT and Gemini), images (eg, Midjourney and DALL·E),^1^ and videos (eg, Sora).^2^ The promising capability of GenAI models are also being explored for their medical applications, such as for mental health,^3^ breast cancer,^4^ dietary care,^5^ and medical education.^6^

However, medical applications of GenAI raise additional ethical concerns due to the ongoing discussions on general AI. For example, the large data volume and user community of LLMs raise the stakes of privacy breaches, as exemplified by a brief ban of ChatGPT in Italy to address and clarify privacy concerns.^7,8^ GenAI-generated data might leak personal information if sufficient details were captured that almost reproduce real training samples.^9^ As GenAI is trained to generate realistic outputs based on patterns learnt from data and can create hallucinations (ie, incorrect and misleading outputs), ensuring factual accuracy for medical applications requires great effort.^10^ Stochasticity of GenAI output, vulnerability to prompt injections, and opacity of input data complicate the assessment of GenAI for clinical fairness, which is predominantly based on predictive performance in the current AI context.^11^ These factors lead to active discussions on, and urgent calls for, new guidelines, regulations, and legislations on GenAI,^12–14^ especially for health care and medical education.^15–17^

Emerging GenAI-powered technology has been quickly rolled out to a huge and diverse user community, which explains why there are enthusiastic discussions on its ethical implications on social media and within the research community. However, other GenAI methods, such as the generative adversarial network (GAN),^18^ have not been considered in current ethical discussions, even though these methods have been used in medical research and are subject to similar ethical considerations. Notably, GAN (and related GenAI methods)^19^ are often used as solutions to address some ethical concerns in medical research (eg, to protect privacy by masking images and videos,^20,21^ or by creating synthetic data).^9,22,23^ A closer investigation of existing discussions on GenAI that cause and mitigate ethical concerns would provide a more holistic view on this important topic, which could ultimately help identify actionable points for GenAI research. In this scoping review, we aimed to understand the responses of the research community to potential ethical issues of GenAI in health care. Informed by these findings, we developed an ethics checklist to operationalise existing ethical guidelines, which can be used by journals, institutional review boards, funders, and regulators to promote responsible GenAI research in health care and beyond.

## Methods

Our systematic scoping review on ethical discussions associated with GenAI in health care followed the PRISMA extension for scoping reviews guidelines.^24^

### Search strategy and selection criteria

We searched PubMed, Embase (Excerpta Medica Database, Ovid), Web of Science, and Scopus for articles written in English with a set of search terms associated with three main concepts: “AI ethics”, “generative AI”, and “healthcare” that were published between Jan 1, 2013, and July 25, 2023. Detailed definitions of the three concepts, search terms, and search strategy are described in the appendix (p 1).

We excluded articles that were not within the domain of health care, did not apply GenAI, or did not discuss ethical issues in relation to the use of GenAI in health care. We also excluded articles that were not peer reviewed, not published as research articles (eg, conference posters, conference abstracts, or book chapters), not full-length (ie, articles citing no more than ten references), or not written in English. The main aim of this scoping review is to understand the current ethical assessments in GenAI research and to inform future practice. Therefore, we restricted our scoping review to the published scientific literature that reflects a reasonable quality of ethical reporting accepted by the research community and that did not include grey literature (eg, preprints).

### Data analysis

The articles we found were divided into three portions and screened based on title and abstract by three pairs of independent reviewers (ST and VN, YN and YS, and YN and DM), following the exclusion criteria. Uncertainties and conflicts were resolved via discussion with ST and YN. The included articles were further screened based on the full text with the same set of exclusion criteria.

From the included articles, we extracted information on seven variables pertaining to article type, GenAI, and ethics: (1) whether the article describes original research (including original empirical and theoretical GenAI research) or review-type articles (eg, reviews, viewpoints, or editorials that specifically covered GenAI); (2) data modalities of GenAI application; (3) GenAI models discussed; (4) role of GenAI (ie, whether it caused or resolved ethical issues); (5) ethical issues discussed; (6) if GenAI caused ethical issues and whether any solution was proposed; and (7) whether the article had ethical objectives or dedicated ethical discussions (as opposed to a brief mention of ethics in background information or general discussion). Based on (7), we further identified a subset of articles with a stronger ethical focus for subsequent checklist development. This subset included review-type articles aiming to discuss GenAI-related ethical issues, and original research either motivated by and framed around specific ethical considerations or discussed detailed ethical implications of GenAI.

To summarise the ethical issues discussed in the included articles, we categorised them through a codification system into nine overarching ethical principles that have been identified to be most pertinent across AI ethics guidelines and in application of AI in health-care settings.^14,25^ We have detailed the adopted definitions (panel 1) and the codes associated with each principle (panel 2), summarised based on the aforementioned discussion on ethics of AI for health care. Due to the focus on ethical concerns on LLMs in research in the past few years, we summarised GenAI application based on three general data modalities, namely text, image (including video), and structured (eg, tabular data, and signal data such as electrocardiogram or speech signal), to investigate any differences in associated ethical considerations. Information on the ethical principles discussed and the role of GenAI by data modality was analysed with evidence gap maps to understand the current research landscape in health care.

### Checklist development

In a 2023 study that proposed an ethical framework of AI for health care for AI developers, the authors endorsed the need to work with health AI practitioners to develop ethical AI checklists as a means to operationalise considerations and solutions to ethical issues.^26^ Hence, to help address prominent gaps in GenAI research, we developed a checklist to promote systematic ethical assessments, named the Transparent Reporting of Ethics for Generative AI (TREGAI) checklist. By analysing the articles identified in our scoping review (especially those with a stronger ethics focus), we selected a set of well established ethical principles in the AI ethics literature to include in our checklist that are essential to, and operationalisable in, GenAI research in health care. We also highlighted the need to discuss solutions to the issues identified. Details on checklist development, specifically the choice of ethical principles and distinction from existing guides on AI, are described in the appendix (p 5).

## Results

**Figure 1:**
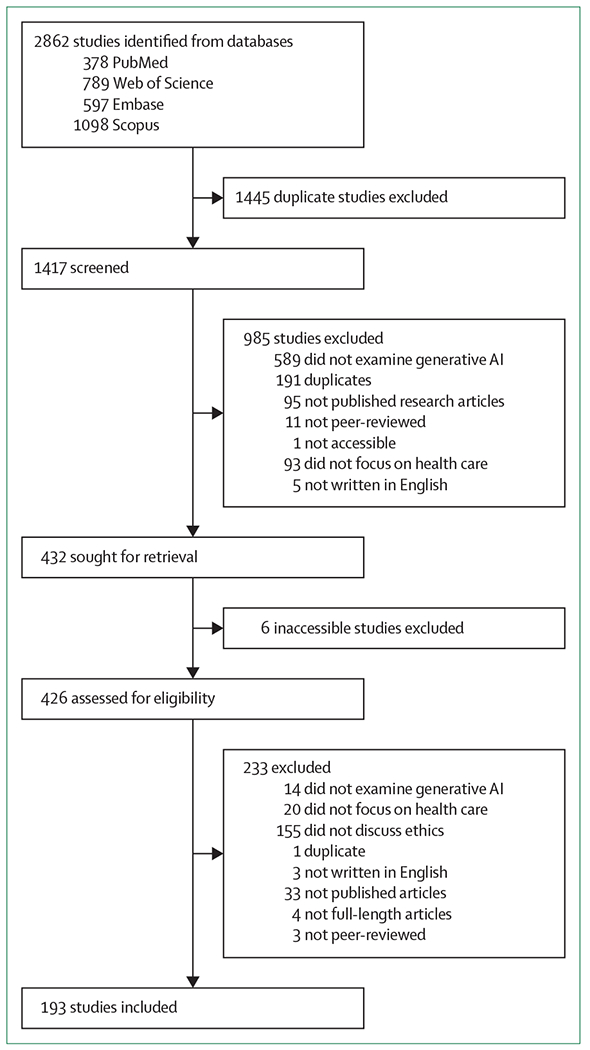
Study selection AI=artificial intelligence.

Our scoping review identified 1417 unique articles, of which 193 articles were included for analysis (figure 1). Detailed information extracted on these articles are presented in the appendix (p 10). The 193 articles included were published between Jan 1, 2018, and July 25, 2023; 162 articles are original research articles and the other 31 articles are review-type articles. 85 articles had dedicated discussions on or quantitative investigations of ethical issues, of which nine original research articles and 20 review-type articles had a stronger ethical focus (appendix p 10).

**Figure 2:**
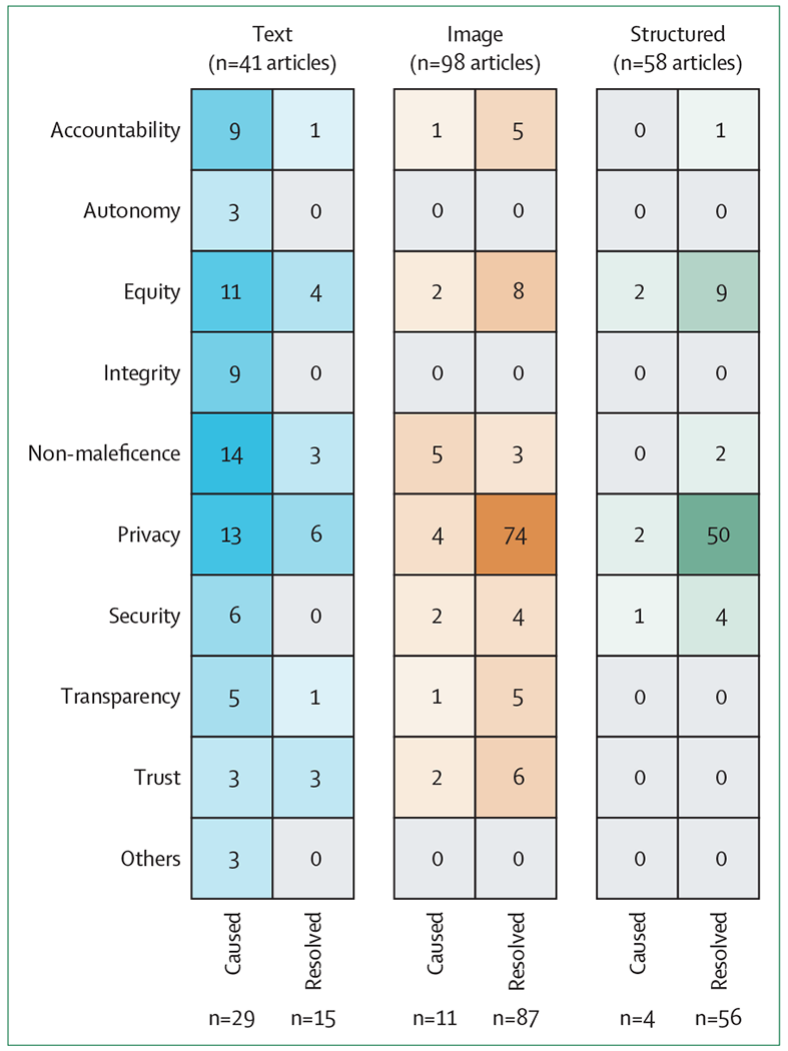
Evidence gap map of ethical issues caused or resolved by generative AI methods for different data modalities Some articles included discussed multiple data modalities and ethical issues caused or resolved by generative AI. An article might discuss both issues caused and resolved by generative AI methods, and might cover multiple ethical issues or data modalities. AI=artificial intelligence.

Although three articles discussed GenAI for more than one data modality (with two articles covering text and image and one article covering text, image, and structured data), most of the articles reviewed were dedicated to a single data modality when discussing ethical issues. Our analysis of these articles revealed notable differences in how researchers approach ethical concerns by data modality, especially on the ethical concerns discussed and the role of GenAI in causing or resolving these concerns (figure 2).

### GenAI for text data-based health care

41 of the 193 articles discussed the ethical considerations of GenAI applications for text data, with 20 articles describing methodological developments or applications of GenAI and the other 21 articles describing review-type work. Although some of these review-type articles used the general term GenAI, the main text and supporting evidence focused on LLMs. 28 articles investigated or had in-depth discussions on ethical issues; the other 13 articles only briefly touched on some ethical aspects.

Among the 41 articles that discussed ethical considerations, 29 focused on the ethical issues caused by LLMs (16 of which focused specifically on generative pretrained transformer models), covering a wide range of application scenarios, and considered the application of all nine ethical principles defined (panel 1; figure 2). Three articles also discussed ethical concerns such as the moral aspects (eg, compassion) of LLM outputs, human–AI interaction, and the rights of LLMs to be considered as coauthors in scientific papers (which we consider as a separate tenth category grouped as other). Of these, one paper only commented briefly on the need for ethical considerations in LLMs. Although all ethical principles are important, some are discussed more often than others, including non-maleficence (also referred to in the literature as benevolence), equity, and privacy, and some articles raised unique concerns related to general AI. In addition to generating medically inaccurate outputs,^5,10^ LLMs can cause harm by partial omission of input information.^4^ Much effort is required to assess the accuracy of LLMs for medical applications, including design of benchmark datasets and manual evaluations.^10^ The ability of LLMs to continuously adapt based on new knowledge raises concerns on the possibility for them to threaten the autonomy of patients and clinicians in medical settings.^27^

The ability of GenAI to work with flexible unstructured input and output provides unique opportunities to mitigate some ethical concerns. 15 of the 41 articles aimed to resolve some existing ethical issues (eg, confidentiality of medical data) by using LLMs and other GenAI (eg, GAN, autoencoder, or a diffusion model) to reduce privacy concerns by generating synthetic medical text, to reduce health-care disparities by providing accessible services and assistance, and to detect health-related misinformation. Specifically, some articles directly assessed human trust on LLMs as these models can generate human-like conversations^28^ or used LLMs to explain AI systems for improved trust and transparency.^29^ Although most articles focused on either identifying ethical issues caused by GenAI or proposing GenAI-based solutions, three articles discussed both to provide a more balanced perspective.

### GenAI for image and structured data-based health care

Unlike the diverse application scenarios of GenAI based on text data, for image and structured data, the use of GenAI focuses on data synthesis and encryption. Hence, most articles discussed the methodological developments of GenAI with a more distinctive and focused set of ethical issues.

Notably, more than half of the articles on image data (63 of 98 articles) and structured data (33 of 58 articles) only mentioned ethical considerations as a brief motivation for methodological developments or as a general discussion point (appendix p 10). The rest of the articles included more in-depth discussions or (mostly quantitative) evaluations of ethical issues. Among these 155 articles (as one article covered multiple modalities), 11 were review-type articles, of which ten articles reviewed methods that mentioned one or two ethical perspectives and only one article^30^ discussed detailed ethical concerns on GenAI applications.

Resolving privacy issues was the main aim of articles for two data modalities (74 articles for image data and 50 articles for structured data; figure 2), predominantly by generating synthetic data with GAN. Eight articles on image data and nine articles on structured data used GenAI to reduce bias (eg, by synthesising data for underrepresented subgroups in existing databases). Similar to LLMs, GenAI contributed to trust and transparency of image classifications by generating realistic images for explanations.^31^ Additionally, GenAI was used to improve accountability of, and hence trust in, image classification by improving input quality.^32^ For both data modalities, we did not see explicit discussions on resolving autonomy or integrity issues with GenAI and for structured data the articles did not have discussions on trust or transparency.

Only 11 articles for image data selectively discussed some of the ethical issues that GenAI can cause, without specific discussions regarding autonomy or integrity (figure 2). For structured data, only four articles discussed equity, privacy, or data security issues caused by GenAI. Only two articles on structured data included both the cause and resolving perspectives, discussing the ethical issues that might arise from limitations of the methods proposed, specifically the risk of biased representation of patient subgroups when synthesising data to resolve privacy issues. As data synthesis is proposed as a solution to medical data deficiency for research due to privacy and security concerns,^22^ multiple articles highlighted the need for data quality and reliability for downstream medical research.^9,22,23,33^ One article introduced metrics to quantify privacy preservation and utility for structured data, but more work is needed to extend for image or text modalities, and to preserve important characteristics of the original data and across small patient subgroups.^34^

## Discussion

Despite the rising number of articles discussing ethical concerns on GenAI in health care, some important aspects are missing in the current literature. Our scoping review systematically summarises the inadequacies in the current literature based on 193 articles, in which we describe the four gaps in current research. Furthermore, we introduce the TREGAI checklist and elaborate on how it might contribute to more responsible GenAI research in health care and in broader application settings.

First, in current GenAI research there are few solutions for ethical issues. For articles focusing on identifying ethical issues caused by GenAI, regulations and guidelines were the most frequently raised solution. This solution was observed in 11 of the 29 articles discussing ethical issues caused by LLMs, and in one article reviewing issues caused by GenAI for images. In total, 16 of the 29 articles on ethical issues caused by LLMs discussed possible solutions, and 11 of these articles mentioned solutions beyond governance, which included ensuring doctor autonomy over LLMs, improving user awareness of the limitations of LLMs, and implementing security technology. The other 13 articles did not discuss any solutions. Although guidelines and regulations for appropriate use of GenAI are important, difficulties can arise when applying ethical guidance based on a set of principles, such as interpreting the specific requirements of a broad ethical principle in any given context of GenAI application or because the guidance offers little assistance when trade-offs need to be made between ethical principles.

Moreover, due to the complexity and fast advancements in methods and technology, compliance to well established legal regulations does not necessarily prevent ethical breaches. For example, the privacy rule enforced by the Health Insurance Portability and Accountability Act of the US Congress is insufficient to prevent privacy breaches (and a few other ethical issues) by the advanced technology used by LLMs;^27^ and additionally, the more recent European AI Act introduced in 2024 is inadequate in aspects such as formal AI definition and risk management.^12^ Similarly, general users of GenAI are unlikely to understand the technology well enough to prescribe a reasonable amount of trust on its output or be able to identify potential misinformation. By contrast, the nonhuman nature and the confident and professional tone of well designed LLMs might earn them unwarranted trust from lay users.^27,35^ Although the magnitude of such influence is not yet clear, the use of LLMs could potentially lead to more privacy leaks and harm from (partly) incorrect or biased information.

Second, discussion on the ethical concerns beyond LLMs is insufficient. Most of the dedicated ethical discussions have focused on LLMs, despite the use of other GenAI methods (eg, GAN) for text data and more prominently for other data modalities. LLM-powered chatbots, such as ChatGPT, make this powerful technology easily accessible to health-care professionals, medical students, and the general public without much need for technical background, hence substantially increasing the impact of any resulting ethical issues. However, similar inadequacies and concerns apply to other GenAI methods and can also affect health care directly or indirectly in the long run. For example, GAN-based approaches have been exploited for insurance scams by editing or injecting fake evidence for diseases into medical images, or have been targeted in cyberattacks to steal confidential information,^36,37^ among other malicious activities, but such topics are more often discussed from purely technical perspectives rather than from ethical perspectives. GAN-based approaches are becoming (if they have not already become) the state-of-the-art models for data synthesis in medical research. However, benchmarks are insufficient to comprehensively assess the quality of synthetic data to preserve privacy and to support unbiased and trustworthy future research,^9,22,23,33,34^ among other ethical gaps (figure 2).

Third, GenAI research does not have a common reference point for ethical discussions. Although viewpoint and perspective articles in leading journals discuss a wide range of ethical issues that could arise (or have arisen) from applications of LLMs in health-care and other settings, articles on GenAI for image or structured data have largely focused on a restricted set of issues, particularly privacy, that could be directly resolved via methodological or technological developments. Ethical discussions can be challenging for GenAI researchers as they are often not formally trained in the definition and interpretation of the ethical principles relevant to AI for health care. However, in the past few years, some studies have included dedicated ethical discussions beyond their main objectives, which shows an increasing interest within the research community to have more in-depth discussions on such issues.^10,38^ Recommendations and guides have been made on the general application of AI for health care,^14,26,39,40^ but currently they are not consistently operationalised in GenAI applications.

On the other hand, different authors might endorse different definitions of ethical terms or might select a subset of ethical keywords for discussion without clear justification. For example, the well established ethical principle of beneficence emphasises improvements to patients’ wellbeing in addition to avoiding harm, but this principle was much less discussed in the literature. Although some ethical principles might be less relevant in some application scenarios than in others (eg, the use of GenAI to synthesise structured data might not have as direct an effect on autonomy as for text data), such statements should be made explicitly by researchers with reasonable justifications rather than being inferred post hoc by users. Incomplete ethical discussions might lead to insufficient methodological developments or questionable applications of existing GenAI methods.

Finally, there is little discussion on multimodal GenAI. Most articles we reviewed involved unimodal GenAI (ie, models that accept and generate data in a single modality). Among the three articles that involved multiple modalities, only two discussed multimodal GenAI (specifically GAN), which simultaneously generated chest x-ray images and radiology reports, and the third article reviewed unimodal GANs for various modalities. Although multimodal GenAI is not yet widely applied in health care,^41^ notable progress has been made in other fields.^42–44^ These extended LLMs will certainly stimulate useful applications in health care and beyond. However, the increased complexity in the models and application settings will further complicate how model reliability is evaluated (which is already challenging for existing LLMs)^45–47^ and the extended capability and wide adoption of such technology can amplify the impact of related ethical issues.

Ongoing research is developing multimodal GenAI from a creative yet concerning approach to reconstruct input to study participants (be it images or text) by analysing their functional MRI (fMRI).^38,48–50^ This approach might be called reverse-mindreading. Each of these methodologies focused on a single input modality, but GenAI trained on one modality can be applied to other modalities with minimal adjustments.^50^ Although these studies provided additional insights on brain functions and potentially some health-care benefits, the direct extraction of information from brain activities beyond health-related purposes poses important concerns in neuroethics.^51,52^ Only two of the four papers explicitly discussed these kinds of ethical concerns, in which one paper performed additional experiments to show preservation of patient privacy^50^ and the other paper only highlighted the general need for regulations.^38^ A more disciplined approach is needed to ensure ethical use of medical data (including but not limited to fMRI) when developing multimodal GenAI for and beyond health-care applications.

To address these gaps in research requires a collaborative effort from researchers, regulators, and stakeholders. Despite the controversies on how to allocate responsibility and credibility to the harm and benefits caused by GenAI,^53^ researchers who develop GenAI (or modify existing ones) should be responsible for understanding and disclosing its capabilities and limitations, and those who apply existing GenAI in their research should be able to justify and discuss its appropriateness and potential issues in context. Some of the articles we reviewed have started call for actions for GenAI developers to reduce ethical issues, such as by highlighting their responsibilities in preventing ethical issues from arising in the first place^16^ and by developing benchmarks to evaluate the ethics of LLMs to facilitate future mitigation,^10^ but such discussions are not easily operationalised in future GenAI research without detailed actionable guides. Hence, we propose to reinforce ethical considerations in GenAI research in health care by mandating standardised and systematic evaluation during peer review via our proposed TREGAI checklist (appendix p 8).

Based on the development of the nine established ethical principles (panel 1) and the additional important principle of beneficence, our suggested TREGAI checklist provides a tool to reinforce systematic ethical assessments for GenAI research. To promote the operationalisation of systematic ethical investigations in research practice, we propose the checklist for use by scientific journals, institutional review boards, funders, and regulators to evaluate ethical discussions in new GenAI research (or proposals). Using the checklist, these users can request researchers to transparently document all ethical issues discussed related to GenAI, provide additional discussions on solutions related to GenAI (ie, solutions to issues caused by GenAI or using GenAI as a solution to ethical issues), and indicate where to find these discussions in the manuscript (appendix p 9). The checklist serves as a reference for peer review by philosophers and ethicists, focusing on the quality of ethical discussions and possible improvements from ethical perspectives. When an ethical principle is deemed not applicable, researchers are strongly encouraged to justify this conclusion within the manuscript. Ethical principles in the checklist vary in the level of abstraction and might not cover all relevant concerns (eg, when working with multimodal GenAI). However, we suggest that journals and other checklist users work with ethicists to adapt the checklist and in-context definitions of ethical principles to their specific needs.

Checklists do have their limitations^54^ and checklist ethics is especially notorious for undermining thorough ethical reflection. Therefore, when using our TREGAI checklist to document ethical discussions, researchers are highly recommended to collaborate with ethicists for in-depth assessments of (potential) ethical concerns and further investigations. By mandating all new research covering GenAI to complete a detailed ethics checklist and undergo peer review of ethical discussions, we at least strive for systematic ethical assessments of GenAI studies that facilitate more responsible and reliable applications. As GenAI is constantly evolving, we maintain the TREGAI checklist live online^55^ to allow timely updates, so that we can incorporate additional ethical principles (eg, from existing literature^26,39^ and emerging multimodal GenAI research^2^), updates in recommended actions, or future progress in GenAI regulations and guidelines to facilitate disciplined, comprehensive, and transparent reinforcement of responsible GenAI research. As the TREGAI checklist focuses on ethical considerations of GenAI, research developing GenAI could use it in addition to existing model development guidelines (eg, TRIPOD^56,57^ or CLAIM^58,59^) and other tools to enhance ethical discussion (eg, the Ethical Operating System Toolkit^60^ and the Organisation for Economic Co-operation and Development ethics principles),^61^ as appropriate. Use of GenAI (eg, ChatGPT) to assist the research process (eg, to draft the manuscript) should refer to the upcoming CANGARU checklist for more specific guides.^62^

With the growing application of AI for health-care and other high-stakes fields, literature is expanding on ethical concerns and guidelines for AI applications.^26,39,63,64^ Similar discussions are extending to GenAI,^16,25,30^ possibly by adopting and expanding ethical frameworks from other domains to health care,^65^ but they are not easily translated into improved research practice.^26^ Our scoping review comprehensively analyses ethical discussions on GenAI in health care to highlight the current absence of a systematic assessment of issues in all relevant application settings (eg, across model types and data modalities) and corresponding solutions, and the reliance on regulations and governance to reinforce ethical standards. However, in view of the complexity of GenAI, ethical considerations need to be incorporated during the development and implementation phase, instead of post-hoc mitigations when issues arise. Hence, we highlight the accountability of researchers to comprehensively discuss, fully disclose, and, when feasible, duly resolve, a well defined set of relevant ethical issues in GenAI research. We develop the TREGAI checklist for use by journals, institutional review boards, funders, regulators, and other potential users as a facilitating tool to promote systematic ethical investigations in GenAI research. As an immediate extension, the TREGAI checklist can be used for ethical assessments of GenAI-powered products derived from research based on user manuals.

As explained in the appendix (p 5), our scoping review might not capture all relevant ethical considerations (eg, sustainability, which is not often discussed in GenAI research for health care). Nonetheless, our scoping review reasonably reflects the current imbalance and inadequacies in ethical discussions on GenAI for health care to inform future research. As an initial step towards more responsible GenAI research, our TREGAI checklist does not include some ethical considerations that are not easily operationalised (eg, morality and dignity), but the checklist can be extended in collaboration with ethicists as appropriate. Moreover, we strongly advocate GenAI researchers to collaborate with ethicists for more in-depth ethical investigations, and, if applicable, modify the definitions of the ethical principles to suit local jurisdictions, culture, and application scenarios. Although the TREGAI checklist targets research-related settings, it could be modified for GenAI-created content (eg, social media posts or teaching materials) to assess benefits, limitations, and potential risks, where the social media platforms and educational institutions might take the responsibility to assemble a team of ethicists to review and advise on ethical issues. By maintaining the TREGAI checklist as a live document online,^55^ we keep it up-to-date with the fast development and expanding applications of GenAI. Meanwhile, additional critical analyses are needed to resolve ethical issues in GenAI research, preferably those which incorporate patients’ perspectives.^66^ Integrating ethical considerations in AI education is another important step towards ethical and responsible future research.^67^

## Conclusion

GenAI is a powerful technology with various potential roles in health care and beyond. Failing to meet ethical standards in these roles can affect daily life by creating inefficient administrative services and can lead to worse health outcomes. However, GenAI is a recent innovation, especially with respect to its use or implementation in medical practice. The number of studies published up until this scoping review is relatively small, but identifying the issues that are essential features of GenAI as early as possible might ultimately help promote trust and adoption for clinicians, patients, and the general public. By suggesting the TREGAI checklist for ethical GenAI and maintaining it live to incorporate updated understanding and regulations, we advocate a systematic and balanced assessment of ethical considerations beyond standard methodological and technological perspectives, which could be extended to general AI and facilitate more responsible and trustworthy development of technology.

## Supplementary Material

1
