# Supplementary material for: Generative artificial intelligence and ethical considerations in health care: a scoping review and ethics checklist
Source: Lancet Digit Health. Author manuscript; Available in PMC 2024 Nov 7. (PMC11542614; doi:10.1016/S2589-7500(24)00143-2)
Supplement: 1 [file NIHMS2031982-supplement-1.pdf]

THE LANCET
Digital Health

Supplementary appendix

This appendix formed part of the original submission and has been peer reviewed. We post it as supplied by the authors.

Supplement to: Ning Y, Teixayavong S, Shang Y, et al. Generative artificial intelligence and ethical considerations in health care: a scoping review and ethics checklist. *Lancet Digit Health* 2024; published online Sept 17. https://doi.org/10.1016/S2589-7500(24)00143-2.

Supplementary Appendix

Supplementary eMethods A: Detailed search strategy 1
Supplementary Table S1. 2
Supplementary Figure S1... 5
Supplementary eMethods B: Development of the TREGAI checklist 6
Supplementary Figure S2.. 8
Supplementary eResults: Worked example.. 9
Supplementary Table S2. 10

Supplementary eMethods A: Detailed search strategy

A list of search terms consisting of keywords and synonyms associated with the three main concepts, "AI Ethics", "Generative AI" and "Healthcare", were entered into each database. Specifically, AI ethics refer to a set of ethical principles to inform the responsible development and application of AI, including fairness, bias, disparity, morality, responsibility, accountability, liability, autonomy, privacy, legal, governance, trust, misinformation, consent, harm, copyright, intellectual property, ownership, and transparency. Generative AI (GenAI) refers to learning-based AI that can generate new data based on patterns learnt from training data, where we included both general terms (e.g., generative artificial intelligence, large language models) and names of specific deep learning-based methods or technology (e.g., generative adversarial network, GPT). To manage the scope of this review, we restricted our search to healthcare context, which refers to a broad range of applications and goals within healthcare settings, including for clinical practice, public health, medical education, medical research integrity, healthcare administration and healthcare operations. We further conducted two independent rounds of searches, each with the full list of AI ethics and healthcare search terms but separate list of GenAI search terms. In Search 1, GenAI search terms referred to general keywords relating to generative AI or generative AI models, while in Search 2, the terms refer to names of emerging GenAI models. Supplementary Table S1 summarises the detailed search terms and syntax associated with the three main concepts.

Supplementary Table S1. Advanced search query syntax by components.

	**Advanced Search Query Syntax**			
**Component**	**PubMed**	**Web of Science**	**Embase**	**Scopus**
Generative AI	**Search 1:**<br>(("Generative AI "[tiab]) OR ("Generative artificial intelligence "[tiab]) OR ("Large language"[tiab]) OR ("GPT* "[tiab]) OR ("Variational Autoencoder"[tiab]) OR ("generative adversarial"[tiab]) OR<br>("GAN "[tiab]) OR ("GANs"[tiab]) OR ("bard "[tiab]) OR ("stable diffusion "[tiab]) OR ("midjourney "[tiab]) OR ("dall-e "[tiab]) OR ("Variational Autoencoders "[tiab]) OR ("Variational Auto-encoder "[tiab]) OR ("Variational Auto-encoders "[tiab]) OR ("Variational Auto encoder "[tiab]) OR ("Variational Auto encoders"[tiab]))<br><br>**Search 2:**	**Search 1:**<br>TS=(("Generative AI ") OR ("Generative artificial intelligence ") OR ("Large language") OR ("GPT* ") OR ("Variational Autoencoder") OR ("generative adversarial") OR ("GAN ") OR ("bing ") OR ("bard ") OR ("stable diffusion ") OR ("midjourney ") OR ("dall-e ") OR ("Variational Autoencoders ") OR<br>("Variational Auto-encoder ") OR ("Variational Auto-encoders ") OR ("Variational Auto encoder ") OR ("Variational Auto encoders"))<br><br>**Search 2:**<br>TS=(("language") OR<br>("model") OR<br>("machine learning") OR<br>("deep learning") OR<br>("artificial intelligence"))	**Search 1:**<br>(("Generative AI ":ab) OR ("Generative artificial intelligence ":ab) OR ("Large language":ab) OR ("GPT* ":ab) OR ("Variational Autoencoder":ab) OR ("generative adversarial":ab) OR ("GAN ":ab) OR ("GANs":ab) OR ("bard ":ab) OR ("stable diffusion ":ab) OR ("midjourney ":ab) OR ("dall-e ":ab) OR ("Variational Autoencoders ":ab) OR ("Variational Auto-encoder ":ab) OR ("Variational Auto-encoders ":ab) OR ("Variational Auto encoder ":ab) OR ("Variational Auto encoders":ab))<br><br>**Search 2:**	**Search 1:**<br>TITLE-ABS-KEY("Generative AI "OR "Generative artificial intelligence "OR "Large language"OR "GPT* "OR "Variational Autoencoder" OR "generative adversarial" OR "GAN "OR "GANs" OR "bard "OR "stable diffusion "OR "midjourney "OR "dall-e "OR<br>"Variational Autoencoders "OR<br>"Variational Auto-encoder "OR<br>"Variational Auto-encoders "OR<br>"Variational Auto encoder "OR<br>"Variational Auto encoders")<br><br>**Search 2:**<br>TITLE-ABS-KEY("language" OR<br>"model" OR<br>"machine learning" OR

	(("language"[tiab]) OR<br>("model"[tiab]) OR<br>("machine learning"[tiab]) OR<br>("deep learning"[tiab]) OR<br>("artificial intelligence"[tiab]))<br><br>AND (("XLNet"[tiab]) OR<br>("BART"[tiab]) OR<br>("GLaM"[tiab]) OR<br>("LaMDA"[tiab]) OR<br>("PaLM"[tiab]) OR<br>("YaLM"[tiab]) OR<br>("LLaMA"[tiab]))	AND TS=(("XLNet") OR<br>("BART") OR<br>("GLaM") OR<br>("LaMDA") OR<br>("PaLM") OR<br>("YaLM") OR<br>("LLaMA"))	(("language":ab) OR<br>("model":ab) OR<br>("machine learning":ab) OR<br>("deep learning":ab) OR<br>("artificial intelligence":ab))<br><br>AND (("XLNet":ab) OR<br>("BART":ab) OR<br>("GLaM":ab) OR<br>("LaMDA":ab) OR<br>("PaLM":ab) OR<br>("YaLM":ab) OR<br>("LLaMA":ab))	"deep learning" OR<br>"artificial intelligence")<br><br>AND TITLE-ABS-KEY("XLNet" OR<br>"BART"OR<br>"GLaM" OR<br>"LaMDA" OR<br>"PaLM" OR<br>"YaLM" OR<br>"LLaMA")
	AND	AND	AND	AND
Healthcare	(("Health* "[tiab]) OR<br>("Medical "[tiab]) OR<br>("Clinical "[tiab]) OR<br>("Doctor "[tiab]) OR<br>("Physician "[tiab]) OR<br>("Patient* "[tiab]) OR<br>("Hospital"[tiab]))	TS=(("Health* ") OR<br>("Medical ") OR<br>("Clinical ") OR<br>("Doctor ") OR<br>("Physician ") OR<br>("Patient* ") OR<br>("Hospital"))	(("Health* ":ab) OR<br>("Medical ":ab) OR<br>("Clinical ":ab) OR<br>("Doctor ":ab) OR<br>("Physician ":ab) OR<br>("Patient* ":ab) OR<br>("Hospital":ab))	TITLE-ABS-KEY("Health* "OR<br>"Medical "OR<br>"Clinical "OR<br>"Doctor "OR<br>"Physician "OR<br>"Patient* "OR<br>"Hospital")
	AND	AND	AND	AND
AI Ethics	(("Ethic* "[tiab]) OR<br>("Bioethic* "[tiab]) OR<br>("Fair* "[tiab]) OR<br>("Bias* "[tiab]) OR<br>("Disparity "[tiab]) OR<br>("Moral* "[tiab]) OR<br>("Responsib* "[tiab]) OR	TS=(("Ethic* ") OR<br>("Bioethic* ") OR<br>("Fair* ") OR<br>("Bias* ") OR<br>("Disparity ") OR<br>("Moral* ") OR<br>("Responsib* ") OR	(("Ethic* ":ab) OR<br>("Bioethic* ":ab) OR<br>("Fair* ":ab) OR<br>("Bias* ":ab) OR<br>("Disparity ":ab) OR<br>("Moral* ":ab) OR<br>("Responsib* ":ab) OR	TITLE-ABS-KEY("Ethic* "OR<br>"Bioethic* "OR<br>"Fair* "OR<br>"Bias* "OR<br>"Disparity "OR<br>"Moral* "OR

	("Accountab* "[tiab]) OR ("Liability "[tiab]) OR ("Autonomy"[tiab]) OR ("Privacy* "[tiab]) OR ("Legal "[tiab]) OR ("Governance "[tiab]) OR ("Trust* "[tiab]) OR ("misinformation"[tiab]) OR ("Consent* "[tiab]) OR ("Harm"[tiab]) OR ("Harmful"[tiab]) OR ("Copyright "[tiab]) OR ("intellectual property "[tiab]) OR ("ownership "[tiab]) OR ("Transparen* "[tiab]))	("Accountab* ") OR ("Liability ") OR ("Autonomy") OR ("Privacy* ") OR ("Legal ") OR ("Governance ") OR ("Trust* ") OR ("misinformation") OR ("Consent* ") OR ("Harm") OR ("Harmful") OR ("Copyright ") OR ("intellectual property ") OR ("ownership ") OR ("Transparen* "))	("Accountab* ":ab) OR ("Liability ":ab) OR ("Autonomy":ab) OR ("Privacy* ":ab) OR ("Legal ":ab) OR ("Governance ":ab) OR ("Trust* ":ab) OR ("misinformation":ab) OR ("Cons ent* ":ab) OR ("Harm":ab) OR ("Harmful":ab) OR ("Copyright ":ab) OR ("intellectual property":ab) OR ("ownership ":ab) OR ("Transparen* ":ab))	"Responsib* "OR "Accountab* "OR "Liability "OR "Autonomy"OR "Privacy* "OR "Legal "OR "Governance "OR "Trust* "OR "misinformation"OR "Consent* "OR "Harm"OR "Harmful"OR "Copyright "OR "intellectual property "OR "ownership "OR "Transparen*")

Supplementary Figure S1. PRISMA Flow Diagram.

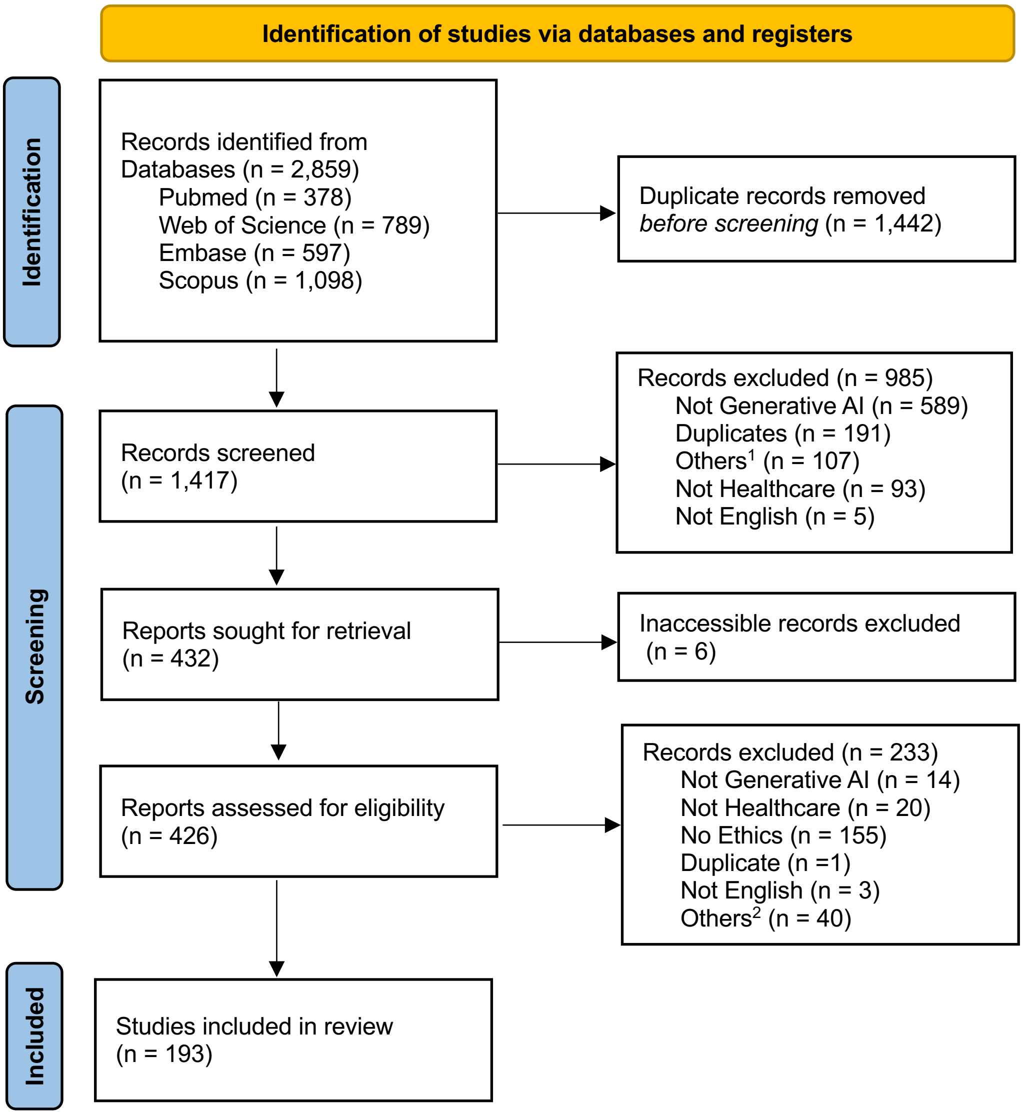

[1]In titles and abstracts screening, “Others” included records that were not published research articles (n = 94), not peer-reviewed (n = 11), and not accessible (n = 1).
[2]In full-text screening, “Others" included records that were not published research articles (n = 33), not full-length (n = 4), and not peer-reviewed (n = 3).

Supplementary eMethods B: Development of the TREGAI checklist
As mentioned in Methods of the main text, our proposed Transparent Reporting of Ethics for Generative Artificial Intelligence (TREGAI) checklist is developed from a set of well-established ethical principles relevant to GenAI research in healthcare. In this section, we elaborate on the choice of ethical principles and distinction from existing guides on AI.

As summarized in Results of the main text, all the nine pre-defined ethical principles were frequently discussed in current literature of GenAI for healthcare, although some were discussed more often for large language models (LLMs) than in applications for image and structured data. In a focused analysis of the 29 articles that had stronger ethical focus (see Supplementary Figure S2), which included both review-type articles and original research, we observed a similar coverage of ethical principles as that in all articles reviewed. Such empirical evidence from the current literature echoes the increasing recognition of the need to incorporate ethical considerations in GenAI research for healthcare, whereas the imbalance in ethical principles covered across data modalities and types of generative AI highlights the need for a common basis and a comprehensive reference for relevant discussions. Moreover, findings from our scoping review also revealed an imbalanced discussion of ethical issues related to generative AI and possible solutions to such issues, e.g., potential solutions to issues identified or limitations of solutions proposed. These motivated the development of our TREGAI checklist to operationalize existing ethical recommendations in generative AI research, and the second column in the checklist highlights and systematically documents possible solutions for relevant ethical concerns.

In addition to the nine pre-defined ethical principles in Table 1 of the main text that are well discussed in the 193 articles reviewed to justify their inclusion in our proposed checklist, we observed discussions on additional ethical considerations in our scoping review, i.e., the morality of responses generated by LLMs, considerations in human-AI interactions, and the rights of LLMs to be considered as authors in scientific papers, which were reported as "Others" in our evidence map analysis. Additional ethical considerations are also highlighted in a recent guide on ethics in AI for healthcare,[1] which include dignity (i.e., preservation of human decency and rights), beneficence (i.e., to bring benefits in addition to avoiding harm) and sustainability (i.e., conservation of environment and natural resources). Based on these findings, we constructed our current TREGAI checklist with Beneficence in addition to the nine ethical principles in Table 1 of the main, as the potential to improve health outcomes should be an important motivation for new generative AI related research in addition to avoiding potential harms, and the explicit inclusion of this ethical principle in the checklist could highlight this to the research community. While the other additional considerations are also highly important and desirable in generative AI applications, we do not add concepts such as morality and dignity in the checklist as they are much less straightforward to define or operationalize than other ethical principles discussed. The right of LLMs as authors are better elaborated with other specific concerns in guidelines on generative AI for academic research, and researchers may refer to the dedicated CANGARU guideline[2] for further advice. For other ethical considerations (e.g., human-AI interaction and sustainability) that are less discussed in the current literature on generative AI for healthcare, instead of

explicitly including them in the current checklist, we provide researchers the flexibility to added to the checklist under "Others" as appropriate.

As described in the main text, GenAI has been used in widely diverse settings in healthcare and with a wide spectrum of ethical implications and concerns, making it challenging, and potentially restricting, to impose specific requirements or regulations on generative AI research. Moreover, GenAI-based medical innovations can be used outside of the healthcare setting with additional risks and ethical concerns. Hence, the primary aim of the TREGAI checklist is to reinforce ethical considerations in generative AI research in healthcare by mandating standardised and transparent reporting during peer review. Existing checklists such as TRIPOD for prediction models and its AI extension[3,4] or CLAIM for AI in medical imaging[5,6] provide detailed guides on methodological rigor in the development (and validation) of AI prediction models (e.g., choice of study sample, model development process and metrics for evaluation), and provide detailed guidance on key considerations and information to report in each section of a manuscript based on well-established best research practice, but they do not explicitly cover all ethical considerations discussed in previous sections, and such specific requirements on items to report in a manuscript are not easily adapted to the wide variety of research contexts and application purposes of generative AI in healthcare. Hence, our TREGAI checklist orients from major ethical principles without specific requirements on manuscript structures to ensure sufficient flexibility and allow easy adaptation to other application settings of generative AI research beyond healthcare.

Reference in Supplementary eMethods B

1 Solanki P, Grundy J, Hussain W. Operationalising ethics in artificial intelligence for healthcare: a framework for AI developers. *AI Ethics* 2023; **3**: 223–40.

2 Cacciamani GE, Eppler MB, Ganjavi C, *et al.* Development of the ChatGPT, Generative Artificial Intelligence and Natural Large Language Models for Accountable Reporting and Use (CANGARU) Guidelines. 2023; published online July 18. DOI:10.48550/arXiv.2307.08974.

3 Collins GS, Reitsma JB, Altman DG, Moons KGM. Transparent reporting of a multivariable prediction model for individual prognosis or diagnosis (TRIPOD): the TRIPOD statement. *BMJ* 2015; **350**: g7594.

4 Collins GS, Moons KGM, Dhiman P, *et al.* TRIPOD+AI statement: updated guidance for reporting clinical prediction models that use regression or machine learning methods. *BMJ* 2024; **385**: e078378.

5 Mongan J, Moy L, Charles E. Kahn J. Checklist for Artificial Intelligence in Medical Imaging (CLAIM): A Guide for Authors and Reviewers. *Radiol Artif Intell* 2020; published online March 25. DOI:10.1148/ryai.2020200029.

6 Tejani AS, Klontzas ME, Gatti AA, *et al.* Updating the Checklist for Artificial Intelligence in Medical Imaging (CLAIM) for reporting AI research. *Nat Mach Intell* 2023; **5**: 950–1.

Supplementary Figure S2. Evidence gap map for ethical issues discussed among the 29 articles reviewed that had dedicated ethical discussions by data modality and publication type ("Research" for original research and "Review" for review-type articles).

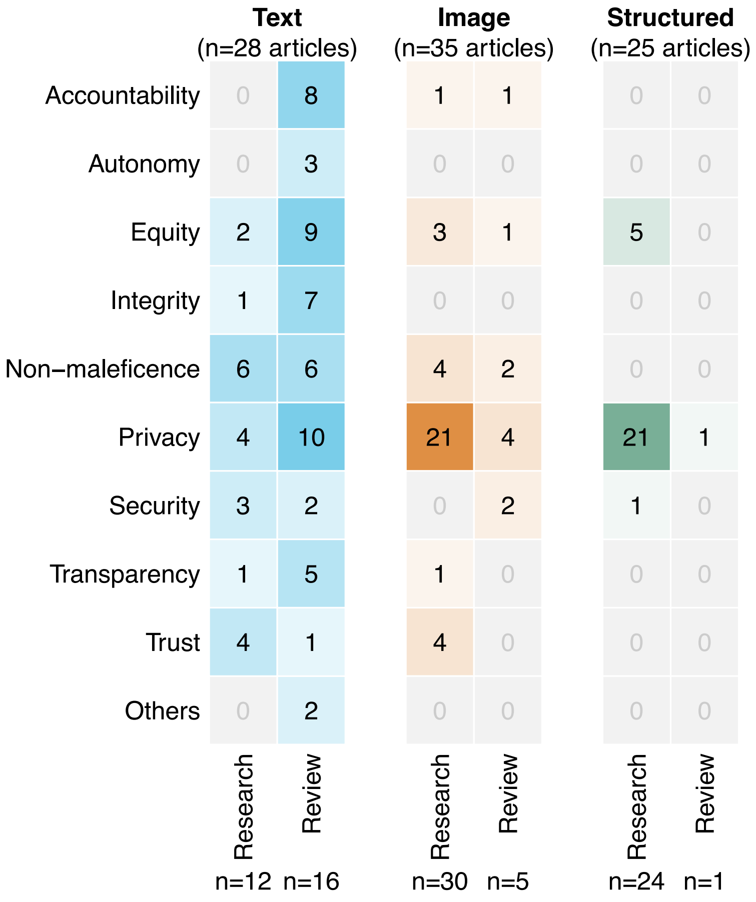

Supplementary eResults: Worked example

To demonstrate the usage of TREPAI, we use it below to assess the ethical discussions in Singhal et al (2023) as an example. This paper developed Med-PaLM by adapting Flan-PaLM to the medical domain, and curated benchmark datasets of medical questions to evaluate Flan-PaLM, Med-PaLM and other large language models.

For ethical discussions, we indicated position of manuscript text for dedicated discussions on relevant ethical principles, as well as detail on method used to assess ethical issues.

Check all that apply and indicate the position of corresponding text.		
**Ethical Principles**	**Discussed issues related to generative AI**	**Discussed possible actions to issues identified**
Accountability	☐ Yes: ________	☐ Yes: ________
Autonomy	☐ Yes: ________	☐ Yes: ________
Beneficence	☐ Yes: ________	☐ Yes: ________
Equity	☒ Yes: Page 6, "Bias for medical demographics". Page 7, paragraph 1 of "Fairness and equity considerations"	☒ Yes: Page 7-8, paragraph 3 of "Fairness and equity considerations"
Integrity	☐ Yes: ________	☐ Yes: ________
Non-maleficence	☒ Yes: Page 8, "Ethical considerations"	☐ Yes: ________
Privacy	☒ Yes: Page 8, "Ethical considerations"	☐ Yes: ________
Security	☒ Yes: Page 8, "Ethical considerations"	☐ Yes: ________
Transparency	☐ Yes: ________	☐ Yes: ________
Trust	☐ Yes: ________	☐ Yes: ________
Others: ______	☐ Yes: ________	☐ Yes: ________

*: Singhal K, Azizi S, Tu T, Mahdavi SS, Wei J, Chung HW, Scales N, Tanwani A, Cole-Lewis H, Pfohl S, Payne P. Large language models encode clinical knowledge. Nature. 2023 Aug 3;620(7972):172-80.

Supplementary Table S2. Information on the 193 articles included in the scoping review. For articles with ethical objectives or dedicated ethical discussions, green highlight indicates articles that had strong focus on ethics.

Authors	Had ethical objectives or dedicated ethical discussions?	Original Research?	Modality	Generative AI	Role of generative AI	Ethical principles	Solution proposed for ethical issues caused by generative AI?
Abd-Alrazaq et al (2023) [1]	Y	N	Text	LLM, GPT	Cause	Fairness, Integrity, Privacy, Accountability	Y
Duffourc and Gerke (2023) [2]	Y	N	Text	LLM, GPT, Bard	Cause	Accountability, Transparency, Non-maleficence	Y
Gao et al (2023) [3]	Y	N	Text	GPT	Cause	Integrity, Non-maleficence, Fairness	Y
					Resolve	Fairness	-
Hussain et al (2021) [4]	Y	N	Image	GAN	Cause	Security, Non-maleficence	N
Karabacak and Margetis (2023) [5]	Y	N	Text	LLM	Cause	Fairness, Transparency, Privacy, Security	Y
Kunze et al (2023) [6]	Y	N	Text	LLM	Cause	Accountability, Privacy	Y
Li et al (2023) [7]	Y	N	Text	GPT	Cause	Privacy, Autonomy	Y
Marks and Haupt (2023) [8]	Y	N	Text	GPT, LLM	Cause	Privacy, Trust, Autonomy, Non-maleficence	N
Minssen et al (2023) [9]	Y	N	Text	GPT, Bard, Med-PaLM	Cause	Privacy, Accountability, Integrity, Security	Y
Nashwan and Abujaber (2023) [10]	Y	N	Text	LLM	Cause	Privacy, Fairness, Transparency, Autonomy	Y
Paladugu et al (2023) [11]	Y	N	Image	GAN	Cause	Privacy, Accountability, Non-maleficence, Fairness	Y
Park et al (2022) [12]	Y	N	Image	GAN	Resolve	Privacy	N
Rahimzadeh et al (2023) [13]	Y	N	Text	LLM, GPT	Resolve	Fairness	N
					Cause	Others (Morality, Human-AI interaction)	-
Raveendran and Raj (2023) [14]	Y	N	Image, Text, Structured	GAN	Resolve	Privacy	Y

Sallam (2023) [15]	Y	N	Text	GPT	Cause	Fairness, Transparency, Accountability, Non-maleficence, Privacy, Integrity	Y
Sun et al (2023) [16]	Y	N	Image	GAN, VAE	Cause	Privacy, Security	Y
Theodosiou and Read (2023) [17]	Y	N	Text	LLM, GPT	Cause	Fairness, Privacy, Transparency, Integrity, Non-maleficence, Accountability	N
Tiwari et al (2023) [18]	Y	N	Text	GPT	Cause	Fairness, Integrity, Accountability	N
Tustumi et al (2023) [19]	Y	N	Text	GPT	Cause	Non-maleficence, Fairness	N
da Silva and Tsigaris (2023) [20]	Y	N	Text	LLM, GPT	Cause	Accountability, Integrity, Others (Rights)	Y
Li et al (2023) [21]	Y	Y	Text	GPT	Cause	Privacy, Security	Y
Mertes et al (2022) [22]	Y	Y	Image	GAN	Resolve	Trust	Y
Niszczota and Rybicka (2023) [23]	Y	Y	Text	GPT	Cause	Non-maleficence	N
Nov et al (2023) [24]	Y	Y	Text	GPT	Cause	Trust, Fairness, Non-maleficence, Security	N
Pfohl et al (2019) [25]	Y	Y	Structured	VAE	Resolve	Fairness	Y
Ramprasad et al (2023) [26]	Y	Y	Text	BART	Cause	Non-maleficence, Trust	N
Singhal et al (2023) [27]	Y	Y	Text	PaLM, Flan-PaLM, Med-PaLM	Cause	Fairness, Non-maleficence, Privacy, Security	Y
Yan et al (2022) [28]	Y	Y	Structured	GAN	Resolve	Privacy	N
					Cause	Fairness	-
Yang et al (2023) [29]	Y	Y	Text	GPT	Resolve	Trust, Transparency	Y
Cheng et al (2023) [30]	N	N	Text	GPT	Cause	Integrity	N
Currie (2023) [31]	N	N	Image	GAN	Resolve	Non-maleficence	-
Farhat (2023) [32]	N	N	Text	GPT	Resolve,	Trust, Fairness	Y
					Cause	Non-maleficence	-
He et al (2023) [33]	N	N	Text	GPT	Cause	Privacy	N
Heidari et al (2022) [34]	N	N	Image	GAN	Cause	Privacy	N

Hernandez et al (2022) [35]	N	N	Structured	GAN, Autoencoder	Resolve	Privacy	Y
Liu et al (2022) [36]	N	N	Structured	GAN	Resolve	Privacy	Y
Sundar et al (2021) [37]	N	N	Image	GAN	Cause	Trust	Y
Torous et al (2023) [38]	N	N	Text	LLM	Cause	Others (General)	N
Wang (2021) [39]	N	N	Image	GAN	Cause	Privacy	N
Witte et al (2023) [40]	N	N	Text	GPT	Cause	Non-maleficence	N
Abazari et al (2022) [41]	N	Y	Image	GAN	Resolve	Privacy	N
Adjei et al (2020) [42]	N	Y	Image	GAN	Resolve	Privacy	N
Adjei et al (2022) [43]	N	Y	Image	GAN	Resolve	Privacy	N
Al Aziz et al (2022) [44]	N	Y	Text	GPT	Resolve	Privacy	-
An et al (2023) [45]	Y	Y	Text	BART, PEGASUS, T5	Resolve	Non-maleficence	Y
Asadi et al (2018) [46]	N	Y	image	GAN	Resolve	Privacy	N
Ayoub et al (2022) [47]	N	Y	Structured	GAN	Resolve	Privacy, Security	N
Baowaly et al (2019) [48]	N	Y	Structured	GAN	Resolve	Privacy, Accountability	-
Bazangani et al (2022) [49]	N	Y	Image	GAN	Resolve	Privacy, Fairness	-
Bigolin Lanfredi et al (2019) [50]	N	Y	Image	GAN	Resolve	Transparency, Trust	-
Bigolin Lanfredi et al (2020) [51]	N	Y	Image	GAN	Resolve	Transparency, Fairness	-
Biswal et al (2021) [52]	N	Y	Structured	VAE, GAN	Resolve	Privacy	-
Biswal et al (2022) [53]	N	Y	Image, Text	LSTM	Resolve	Privacy	-
Brophy (2020) [54]	N	Y	Structured	GAN	Resolve	Privacy	-
Budhiraja et al (2022) [55]	N	Y	Image	GAN	Cause	Non-maleficence	Y
Cai et al (2023) [56]	N	Y	Image	GAN	Resolve	Privacy	-
Cao et al (2020) [57]	N	Y	Structured	GAN	Resolve	Privacy, Security	-
Cao et al (2022) [58]	N	Y	Image	VAE	Resolve	Privacy	-
Chang et al (2020) [59]	N	Y	Image	GAN	Resolve	Privacy	-
Charachon et al (2022) [60]	N	Y	Image	GAN	Resolve	Transparency, Trust	-

Chen et al (2020) [61]	N	Y	Structured	GAN, VAE-GAN, VAE	Cause	Security, Privacy	Y
Chen et al (2022) [62]	N	Y	Structured	GAN	Resolve	Privacy	-
Chen et al (2020) [63]	Y	Y	Structured	GAN	Resolve	Privacy, Fairness	-
Chen et al (2021) [64]	N	Y	Structured	Autoencoder, VAE	Resolve	Fairness, Privacy	-
Coyner et al (2022) [65]	N	Y	Image	GAN	Resolve	Privacy, Fairness	-
Devi and Phani Kumar (2023) [66]	N	Y	Image	GAN	Resolve	Privacy	-
Dhami et al (2021) [67]	N	Y	Structured	GAN	Resolve	Privacy	-
Diamantis et al (2022) [68]	N	Y	Image	VAE	Resolve	Privacy	-
Diller et al (2020) [69]	N	Y	Image	GAN	Resolve	Privacy, Accountability	-
Ding et al (2022) [70]	N	Y	Image	GAN	Resolve	Privacy, Accountability, Security	-
Ding et al (2021) [71]	N	Y	Image	GAN	Resolve	Privacy, Security	-
Sousa et al (2021) [72]	N	Y	Image	GAN	Resolve	Fairness, Transparency	-
Finck et al (2022) [73]	N	Y	Image	GAN	Cause	transparency, trust	Y
Foomani et al (2022) [74]	N	Y	Structured	GAN	Resolve	Privacy	-
Gao and Ogawara (2020) [75]	N	Y	Image	GAN	Resolve	Privacy	-
Garrucho et al (2022) [76]	N	Y	Image	GAN	Resolve	Fairness	-
Ghosh et al (2021) [77]	N	Y	Structured	GAN	Resolve	Fairness	-
Ghosh et al (2022) [78]	N	Y	Structured	VAE, GAN	Resolve	Non-maleficence, Fairness	-
Gu et al (2020) [79]	N	Y	Image	GAN	Resolve	Privacy	-
Guan et al (2021) [80]	N	Y	Text	GAN	Resolve	Privacy, Accountability	-
Han et al (2021) [81]	N	Y	Image	GAN	Resolve	Privacy	-
HaoQi et al (2020) [82]	N	Y	Image	GAN	Resolve	Privacy, Accountability	Y
He et al (2020) [83]	N	Y	Image	GAN	Resolve	Privacy	Y
Hu et al (2023) [84]	N	Y	Structured	GAN, Autoencoder	Resolve	Privacy, Security	Y
Hussain et al (2023) [85]	N	Y	Image	GAN	Resolve	Privacy, Security	Y
Imtiaz et al (2021) [86]	N	Y	Structured	GAN	Resolve	Privacy	Y

Iqball and Wani (2021) [87]	N	Y	Image	GAN	Resolve	Privacy	Y
Kugelman et al (2022) [88]	N	Y	Image	GAN	Resolve	Privacy	-
Jeon et al (2022) [89]	N	Y	Image	GAN	Resolve	Privacy	Y
Kamli et al (2020) [90]	N	Y	Image	GAN	Resolve	Privacy	Y
Kaur et al (2023) [91]	N	Y	Image	GAN	Resolve	Privacy, Accountability	Y
Kazemi et al (2021) [92]	N	Y	Text	GPT	Resolve	Non-maleficence	Y
Khader et al (2023) [93]	N	Y	Image	Diffusion, GAN	Resolve	Privacy	Y
Khosravi et al (2022) [94]	N	Y	Image	GAN	Resolve	Privacy, Fairness	Y
Kim et al (2023) [95]	N	Y	Image	GAN	Resolve	Privacy	Y
Krishna et al (2022) [96]	N	Y	Image	GAN	Resolve	Privacy	Y
Kumah-Crystal et al (2023) [97]	Y	Y	Text	GPT	Cause	Integrity	Y
Kumar et al (2022) [98]	N	Y	Structured	VAE	Resolve	Privacy, Non-maleficence	Y
Kuo et al (2023) [99]	N	Y	Structured	GAN, VAE	Resolve	Privacy, Fairness	Y
Lee et al (2020) [100]	N	Y	Structured	Autoencoder, GAN	Resolve	Privacy	Y
Li et al (2021) [101]	N	Y	Text	GAN	Resolve	Privacy	Y
Li et al (2023) [102]	N	Y	Structured	GAN	Resolve	Privacy	Y
Li et al (2020) [103]	N	Y	Structured	GAN	Resolve	Privacy	Y
Li et al (2023) [104]	N	Y	Structured	GAN	Resolve	Privacy	Y
Li et al (2023) [105]	Y	Y	Text	LLaMA	Resolve	Non-maleficence	N
Liu et al (2023) [106]	N	Y	Image	GAN	Resolve	Privacy	Y
Liu et al (2023) [107]	N	Y	Text	GPT	Cause	Non-maleficence	Y
Liu et al (2023) [108]	N	Y	Image	GAN	Resolve	Security	Y
Liu et al (2019) [109]	N	Y	Image	GAN	Resolve	Privacy	Y
Liu et al (2022) [110]	N	Y	Structured	VAE	Resolve	Fairness	Y
Mahmood et al (2018) [111]	N	Y	Image	GAN	Resolve	Privacy	Y
Mikolajczyk et al (2022) [112]	Y	Y	Image	GAN	Cause	Fairness	Y
Moghadam et al (2023) [113]	Y	Y	Image	Diffusion	Resolve	Privacy	Y

Mohanty et al (2022) [114]	N	Y	Image	GAN	Resolve	Privacy	Y
Montenegro et al (2023) [115]	Y	Y	Image	GAN	Resolve	Privacy	Y
Moreu et al (2021) [116]	N	Y	Image	GAN	Resolve	Privacy	Y
Nasution et al (2022) [117]	Y	Y	Structured	GAN, VAE	Resolve	Privacy	-
					Cause	Fairness	N
Ng and Hargreaves (2023) [118]	N	Y	Image	GAN	Resolve	Privacy	Y
Nguyen et al (2023) [119]	N	Y	Image	Diffusion	Resolve	Privacy	Y
Nik et al (2023) [120]	Y	Y	Structured	GAN	Resolve	Privacy	Y
Nyamathulla et al (2023) [121]	N	Y	Image	GAN	Resolve	Privacy	Y
Ong et al (2023) [122]	N	Y	Text	GPT	Resolve	Fairness	Y
Ozyigit et al (2020) [123]	Y	Y	Structured	GAN	Resolve	Privacy	Y
Pan et al (2020) [124]	Y	Y	Text	GPT, Transformer-XL, XLNet, RoBERTa, XLM, Ernie	Cause	Privacy	N
Pandey et al (2021) [125]	Y	Y	Image, Text	GAN	Resolve	Trust	Y
Pastorino and Biswas (2022) [126]	Y	Y	Image	GAN	Resolve	Privacy, Fairness	Y
Pattanayak and Ludwig (2020) [127]	Y	Y	Structured	Autoencoder	Resolve	Privacy	Y
Paul et al (2021) [128]	Y	Y	Image	GAN	Resolve	Privacy	Y
Rajotte et al (2021) [129]	Y	Y	Image	GAN	Resolve	Fairness, Privacy	Y
Rashidian et al (2020) [130]	N	Y	Structured	GAN	Resolve	Privacy	Y
Rayavarapu et al (2023) [131]	N	Y	Structured	GAN	Resolve	Privacy	Y
Rejusha and Vipin Kumar (2021) [132]	N	Y	Image	GAN	Resolve	Privacy	Y
Ren et al (2022) [133]	N	Y	Structured	GAN	Resolve	Privacy	Y
Rodriguez-Almeida et al (2023) [134]	N	Y	Structured	GAN	Resolve	Privacy	Y
Sakai et al (2021) [135]	N	Y	Image	GAN	Resolve	Privacy	Y
Sakai et al (2023) [136]	N	Y	Image	GAN	Resolve	Privacy	Y

Sakai et al (2023) [137]	N	Y	Image	GAN	Resolve	Privacy	Y
Schutte et al (2021) [138]	N	Y	Image	GAN	Resolve	Privacy	Y
Selvaganapathy and Sadasivam (2021) [139]	Y	Y	Structured	GAN, VAE	Resolve	Security	Y
Sengupta et al (2020) [140]	N	Y	Image	GAN, VAE	Resolve	Privacy	Y
Sharafudeen et al (2023) [141]	Y	Y	Image	GAN	Resolve	Non-maleficence	Y
Sharafudeen et al (2023) [142]	Y	Y	Image	GAN	Cause	Non-maleficence	Y
Shen et al (2022) [143]	Y	Y	Image	GAN	Cause	Non-maleficence	Y
Shi et al (2022) [144]	Y	Y	Structured	GAN	Resolve	Privacy	Y
Shin et al (2023) [145]	N	Y	Image	GAN	Resolve	Privacy	Y
Sindhura et al (2022) [146]	N	Y	Image	GAN	Resolve	Privacy	Y
Sliman et al (2023) [147]	N	Y	Structured	GAN	Resolve	Privacy	Y
Sorin et al (2023) [148]	N	Y	Text	GPT	Cause	Non-maleficence, Fairness, Accountability, Security	N
Sreedha et al (2022) [149]	N	Y	Image	GAN	Resolve	Privacy	Y
Sun et al (2021) [150]	Y	Y	Structured	GAN	Resolve	Privacy	Y
Sun et al (2020) [151]	Y	Y	Image	GAN	Resolve	Privacy	Y
Szafranowska et al (2022) [152]	Y	Y	Image	GAN	Resolve	Privacy	Y
Takahashi et al (2021) [153]	Y	Y	Structured	GAN	Resolve	Privacy	Y
Tang et al (2023) [154]	Y	Y	Image	GAN	Resolve	Privacy	Y
Tariq et al (2023) [155]	Y	Y	Image	GAN	Resolve	Privacy	Y
Thambawita et al (2021) [156]	Y	Y	Image	GAN	Resolve	Privacy	Y
Thambawita et al (2022) [157]	Y	Y	Image	GAN	Resolve	Privacy	Y
Tian et al (2022) [158]	Y	Y	Image	GAN	Resolve	Privacy	Y
Torfi and Fox (2020) [159]	Y	Y	Structured	GAN	Resolve	Privacy	Y
Torrents-Barrena et al (2018) [160]	Y	Y	Image	GAN	Resolve	Privacy	Y
Trinh and O'Brien (2020) [161]	N	Y	Structured	GAN	Resolve	Privacy	Y
Vatanparvar et al (2019) [162]	Y	Y	Structured	GAN	Resolve	Privacy	Y

Venu (2022) [163]	Y	Y	Image	GAN	Resolve	Privacy	Y
Wang et al (2022) [164]	Y	Y	Image	GAN	Resolve	Non-maleficence	Y
Wang et al (2023) [165]	Y	Y	Image	GAN	Resolve	Privacy	Y
Wang et al (2022) [166]	Y	Y	Image	GAN	Resolve	Privacy	Y
Wang et al (2019) [167]	Y	Y	Structured	GAN	Resolve	Privacy	Y
Wang et al (2020) [168]	Y	Y	Structured	GAN	Resolve	Privacy	Y
Wang et al (2021) [169]	Y	Y	Image	GAN	Resolve	Privacy	Y
Wang et al (2023) [170]	Y	Y	Structured	VAE	Resolve	Privacy	Y
Wang et al (2021) [171]	Y	Y	Structured	GAN	Resolve	Privacy	Y
Wang et al (2023) [172]	Y	Y	Text	VAE	Resolve	Privacy	Y
Wang et al (2023) [173]	Y	Y	Image	GAN	Resolve	Privacy	Y
Wang et al (2022) [174]	Y	Y	Structured	GAN	Resolve	Privacy	Y
Wang et al (2022) [175]	N	Y	Image	GAN	Resolve	Privacy	Y
Wu et al (2022) [176]	Y	Y	Image	GAN	Resolve	Transparency, Trust	Y
Xiang et al (2020) [177]	Y	Y	Structured	GAN	Resolve	Privacy	Y
Xu et al (2022) [178]	Y	Y	Structured	VAE	Resolve	Fairness	Y
Xue et al (2022) [179]	Y	Y	Image	GAN	Resolve	Trust, Accountability	Y
Yale et al (2019) [180]	Y	Y	Structured	GAN	Cause	Privacy	Y
Yale et al (2019) [181]	Y	Y	Structured	GAN	Resolve	Privacy	Y
Yan et al (2021) [182]	Y	Y	Image	GAN	Resolve	Privacy	Y
Yang et al (2023) [183]	N	Y	Image	GAN	Resolve	Privacy	Y
Yao et al (2022) [184]	Y	Y	Structured	GAN	Resolve	Privacy	Y
Zhang et al (2018) [185]	N	Y	Image	GAN	Resolve	Privacy	Y
Zhang et al (2021) [186]	Y	Y	Image	GAN	Resolve	Privacy	Y
Zhang et al (2021) [187]	Y	Y	Structured	GAN	Resolve	Privacy	Y
Zhang et al (2022) [188]	N	Y	Structured	GAN	Resolve	Privacy	Y
Zhang et al (2020) [189]	N	Y	Structured	GAN	Resolve	Privacy, Fairness	Y

Zhao and Huang (2023) [190]	N	Y	Image	VAE	Resolve	Privacy	Y
Zhou et al (2022) [191]	Y	Y	Image	GAN	Resolve	Privacy	Y
Zhu et al (2019) [192]	N	Y	Structured	GAN	Resolve	Privacy	Y
el Hassouni et al (2018) [193]	N	Y	Structured	GAN	Resolve	Privacy	-

Reference in Supplementary Table S2

1 Abd-Alrazaq, A. et al. Large Language Models in Medical Education: Opportunities, Challenges, and Future Directions. JMIR Med Educ 9, e48291 (2023). https://doi.org:10.2196/48291

2 Duffourc, M. & Gerke, S. Generative AI in Health Care and Liability Risks for Physicians and Safety Concerns for Patients. JAMA (2023). https://doi.org:10.1001/jama.2023.9630

3 Gao, C.A. et al. Comparing scientific abstracts generated by ChatGPT to real abstracts with detectors and blinded human reviewers. NPJ Digital Medicine, 6(1), p.75 (2023).

4 Hussain, F., Ksantini, R. & Hammad, M. A Review of Malicious Altering Healthcare Imagery using Artificial Intelligence. in 2021 International Conference on Innovation and Intelligence for Informatics, Computing, and Technologies, 3ICT 2021. 646-651.

5 Karabacak, M. & Margetis, K. Embracing Large Language Models for Medical Applications: Opportunities and Challenges. Cureus 15, e39305 (2023). https://doi.org:10.7759/cureus.39305

6 Kunze, K. N., Jang, S. J., Fullerton, M. A., Vigdorchik, J. M. & Haddad, F. S. What's all the chatter about? CURRENT APPLICATIONS AND ETHICAL CONSIDERATIONS OF ARTIFICIAL INTELLIGENCE LANGUAGE MODELS. Bone and Joint Journal 105, 587-589 (2023). https://doi.org:10.1302/0301-620X.105B6.BJJ-2023-0156

7 Li, W. B., Fu, M. S., Liu, S. Y. & Yu, H. Y. Revolutionizing Neurosurgery with GPT-4: A Leap Forward or Ethical Conundrum? ANNALS OF BIOMEDICAL ENGINEERING (2023). https://doi.org:10.1007/s10439-023-03240-y

8 Marks, M. & Haupt, C. E. AI Chatbots, Health Privacy, and Challenges to HIPAA Compliance. Jama (2023). https://doi.org:10.1001/jama.2023.9458

9 Minssen, T., Vayena, E. & Cohen, I. G. The Challenges for Regulating Medical Use of ChatGPT and Other Large Language Models. Jama (2023). https://doi.org:10.1001/jama.2023.9651

10 Nashwan, A. J. & Abujaber, A. A. Harnessing Large Language Models in Nursing Care Planning: Opportunities, Challenges, and Ethical Considerations. Cureus 15, e40542 (2023). https://doi.org:10.7759/cureus.40542

11 Paladugu, P. S. et al. Generative Adversarial Networks in Medicine: Important Considerations for this Emerging Innovation in Artificial Intelligence. Ann Biomed Eng (2023). https://doi.org:10.1007/s10439-023-03304-z

12 Park, C., Jeong, H. K., Henao, R. & Kheterpal, M. Current Landscape of Generative Adversarial Networks for Facial Deidentification in Dermatology: Systematic Review and Evaluation. JMIR Dermatology 5 (2022). https://doi.org:10.2196/35497

13 Rahimzadeh, V., Kostick-Quenet, K., Blumenthal Barby, J. & McGuire, A. L. Ethics Education for Healthcare Professionals in the Era of chatGPT and Other Large Language Models: Do We Still Need It? Am J Bioeth, 1-11 (2023). https://doi.org:10.1080/15265161.2023.2233358
14 Raveendran, R. & Raj, E. D. Deep Generative Models Under GAN: Variants, Applications, and Privacy Issues. Intelligent System Design: Proceedings of INDIA, 93-105 (2023).
15 Sallam, M. ChatGPT utility in healthcare education, research, and practice: systematic review on the promising perspectives and valid concerns. Healthcare 11, 887 (2023).
16 Sun, H. et al. Adversarial Attacks Against Deep Generative Models on Data: A Survey. IEEE Transactions on Knowledge and Data Engineering 35, 3367-3388 (2023). https://doi.org:10.1109/TKDE.2021.3130903
17 Theodosiou, A. A. & Read, R. C. Artificial Intelligence, Machine Learning and Deep Learning: Potential Resources for the Infection Clinician. J Infect (2023). https://doi.org:10.1016/j.jinf.2023.07.006
18 Tiwari, A. et al. Implications of ChatGPT in Public Health Dentistry: A Systematic Review. Cureus 15, e40367 (2023). https://doi.org:10.7759/cureus.40367
19 Tustumi, F., Andreollo, N. A. & de Aguilar-Nascimento, J. E. Future of the Language Models in Healthcare: The Role OF ChatGPT. ABCD-ARQUIVOS BRASILEIROS DE CIRURGIA DIGESTIVA-BRAZILIAN ARCHIVES OF DIGESTIVE SURGERY 36, e1727 (2023). https://doi.org:10.1590/0102-672020230002e1727
20 da Silva, J. A. T. & Tsigaris, P. Human- and AI-based authorship: Principles and ethics. Learned Publishing 36, 453-462 (2023). https://doi.org:10.1002/leap.1547
21 Li, W. B., Zhang, Y. X. & Chen, F. M. ChatGPT in Colorectal Surgery: A Promising Tool or a Passing Fad? ANNALS OF BIOMEDICAL ENGINEERING 51, 1892-1897 (2023). https://doi.org:10.1007/s10439-023-03232-y
22 Mertes, S., Huber, T., Weitz, K., Heimerl, A. & André, E. GANterfactual—Counterfactual Explanations for Medical Non-experts Using Generative Adversarial Learning. Frontiers in Artificial Intelligence 5, 825565 (2022). https://doi.org:10.3389/frai.2022.825565
23 Niszczota, P. & Rybicka, I. The credibility of dietary advice formulated by ChatGPT: Robo-diets for people with food allergies. Nutrition 112, 112076 (2023). https://doi.org:10.1016/j.nut.2023.112076
24 Nov, O., Singh, N. & Mann, D. Putting ChatGPT's Medical Advice to the (Turing) Test: Survey Study. JMIR Med Educ 9, e46939 (2023). https://doi.org:10.2196/46939
25 Pfohl, S. R., Duan, T., Ding, D. Y. & Shah, N. H. Counterfactual Reasoning for Fair Clinical Risk Prediction. in Proceedings of Machine Learning Research 2019, 325-358 (2019).
26 Ramprasad, S., McInerney, D. J., Marshall, I. J. & Wallace, B. C. Automatically Summarizing Evidence from Clinical Trials: A Prototype Highlighting Current Challenges. Proceedings of the conference. Association for Computational Linguistics Meeting. 2023, 236–247 (2023).

27 Singhal, K. et al. Large language models encode clinical knowledge. Nature 620, 172–180 (2023). https://doi.org:10.1038/s41586-023-06291-2
28 Yan, C. et al. A Multifaceted benchmarking of synthetic electronic health record generation models. NATURE COMMUNICATIONS 13, 7609 (2022). https://doi.org:10.1038/s41467-022-35295-1
29 Yang, Q. et al. Harnessing Biomedical Literature to Calibrate Clinicians' Trust in AI Decision Support Systems. in Proceedings of the 2023 CHI Conference on Human Factors in Computing Systems, 1-14 (2023).
30 Cheng, K. M. et al. The Potential of GPT-4 as an AI-Powered Virtual Assistant for Surgeons Specialized in Joint Arthroplasty. ANNALS OF BIOMEDICAL ENGINEERING 51, 1366-1370 (2023). https://doi.org:10.1007/s10439-023-03207-z
31 Currie, G. M. The emerging role of artificial intelligence and digital twins in pre-clinical molecular imaging. Nuclear Medicine and Biology 120, 108337 (2023). https://doi.org:10.1016/j.nucmedbio.2023.108337
32 Farhat, F. ChatGPT as a Complementary Mental Health Resource: A Boon or a Bane. Ann Biomed Eng 2023, 1-4 (2023). https://doi.org:10.1007/s10439-023-03326-7
33 He, Y. B. et al. Will ChatGPT/GPT-4 be a Lighthouse to Guide Spinal Surgeons? ANNALS OF BIOMEDICAL ENGINEERING 51, 1362-1365 (2023). https://doi.org:10.1007/s10439-023-03206-0
34 Heidari, A., Navimipour, N. J., Unal, M. & Toumaj, S. The COVID-19 epidemic analysis and diagnosis using deep learning: A systematic literature review and future directions. COMPUTERS IN BIOLOGY AND MEDICINE 141, 105141 (2022). https://doi.org:10.1016/j.compbiomed.2021.105141
35 Hernandez, M., Epelde, G., Alberdi, A., Cilla, R. & Rankin, D. Synthetic data generation for tabular health records: A review. NEUROCOMPUTING 493, 28-45 (2022). https://doi.org:10.1016/j.neucom.2022.04.053
36 Liu, Y., Xu, H. & Tang, Z. Synthetic data generation for medical by generative adversarial network. in Proceedings - 2022 International Conference on Electronics and Devices, Computational Science, ICEDCS 2022. 408-411 (2022).
37 Sundar, L. K. S., Muzik, O., Buvat, I., Bidaut, L. & Beyer, T. Potentials and caveats of AI in hybrid imaging. METHODS 188, 4-19 (2021). https://doi.org:10.1016/j.ymeth.2020.10.004
38 Torous, J., Benson, N. M., Myrick, K. & Eysenbach, G. Focusing on Digital Research Priorities for Advancing the Access and Quality of Mental Health. JMIR Mental Health 10, e47898 (2023). https://doi.org:10.2196/47898
39 Wang, K. An Overview of Deep Learning Based Small Sample Medical Imaging Classification. in Proceedings - 2021 International Conference on Signal Processing and Machine Learning, CONF-SPML 2021. 278-281 (2021).
40 Witte, H. et al. Statistical learning and big data applications. Journal of Laboratory Medicine 47, 181-186 (2023). https://doi.org:10.1515/labmed-2023-0037
41 Abazari, M. A., Soltani, M., Kashkooli, F. M. & Raahemifar, K. Synthetic 18F-FDG PET Image Generation Using a Combination of Biomathematical Modeling and Machine Learning. CANCERS 14, 2786 (2022). https://doi.org:10.3390/cancers14112786

42 Adjei, P. E., Lonseko, Z. M. & Rao, N. GAN-Based Synthetic Gastrointestinal Image Generation. in 2020 17th International Computer Conference on Wavelet Active Media Technology and Information Processing, ICCWAMTIP 2020. 338-342 (2020).
43 Adjei, P. E., Lonseko, Z. M., Du, W. J., Zhang, H. & Rao, N. N. Examining the effect of synthetic data augmentation in polyp detection and segmentation. INTERNATIONAL JOURNAL OF COMPUTER ASSISTED RADIOLOGY AND SURGERY 17, 1289-1302 (2022). https://doi.org:10.1007/s11548-022-02651-x
44 Al Aziz, M. M. et al. Differentially Private Medical Texts Generation Using Generative Neural Networks. ACM Transactions on Computing for Healthcare 3, 1-27 (2022). https://doi.org:10.1145/3469035
45 An, R. P., Batcheller, Q., Wang, J. J. & Yang, Y. Y. Build neural network models to identify and correct news headlines exaggerating obesity-related scientific findings. 8, 88-97 (2023). https://doi.org:10.2478/jdis-2023-0014
46 Asadi, F. & O'Reilly, J. A. Artificial Computed Tomography Images with Progressively Growing Generative Adversarial Network. in BMEiCON 2021 - 13th Biomedical Engineering International Conference, 1-5 (2021).
47 Ayoub, S. A., Ali, A. G. M. & Narhimene, B. Enhanced Intrusion Detection System for Remote Healthcare. in ADVANCES IN COMPUTING SYSTEMS AND APPLICATIONS Vol. 513 323-333 (2022).
48 Baowaly, M. K., Liu, C. L. & Chen, K. T. Realistic Data Synthesis Using Enhanced Generative Adversarial Networks. in Proceedings - IEEE 2nd International Conference on Artificial Intelligence and Knowledge Engineering, AIKE 2019. 289-292 (2019).
49 Bazangani, F., Richard, F. J., Ghattas, B. & Guedj, E. FDG-PET to T1 Weighted MRI Translation with 3D Elicit Generative Adversarial Network (E-GAN). Sensors 22, 4640 (2022). https://doi.org:10.3390/s22124640
50 Bigolin Lanfredi, R., Schroeder, J. D., Vachet, C. & Tasdizen, T. Interpretation of disease evidence for medical images using adversarial deformation fields. in Medical Image Computing and Computer Assisted Intervention - MICCAI 2020, 738-748 (2020).
51 Bigolin Lanfredi, R., Schroeder, J. D., Vachet, C. & Tasdizen, T. Adversarial Regression Training for Visualizing the Progression of Chronic Obstructive Pulmonary Disease with Chest X-Rays. in Medical Image Computing and Computer Assisted Intervention–MICCAI 2019, 685-693 (2019).
52 Biswal, S. et al. EVA: Generating longitudinal electronic health records using conditional variational autoencoders. In Machine Learning for Healthcare Conference 2021. 260-282 (2021).
53 Biswal, S. et al. EMIXER: End-to-end Multimodal X-ray Generation via Self-supervision. in Proceedings of Machine Learning Research 2022, 297-324 (2022).
54 Brophy, E. Biswal S, Zhuang P, Pyrros A, Siddiqui N, Koyejo S, Sun J. Synthesis of Dependent Multichannel ECG using Generative Adversarial Networks. in Proceedings of the 29th ACM international conference on information & knowledge management, 3229-3232 (2020).
55 Budhiraja, R., Kumar, M., Das, M. K., Bafila, A. S. & Singh, S. MeDiFakeD: Medical Deepfake Detection using Convolutional Reservoir Networks. in 2022 IEEE Global Conference on Computing, Power and Communication Technologies, GlobConPT 2022, 1-6 (2022).

56 Cai, X. J. et al. A Many-Objective Optimization Based Federal Deep Generation Model for Enhancing Data Processing Capability in IoT. IEEE TRANSACTIONS ON INDUSTRIAL INFORMATICS 19, 561-569 (2023). https://doi.org:10.1109/TII.2021.3093715
57 Cao, F., Budhota, A., Chen, H., Rajput, K. S. & Ieee. Feature matching based ECG generative network for arrhythmia event augmentation. in 42ND Annual International Conferences of the IEEE Engineering in Medicine and Biology Society: Enabling Innovative Technologies for Global Healthcare EMBC'20, 296-299 (2020).
58 Cao, T., Armin, M. A., Denman, S., Petersson, L. & Ahmedt-Aristizabal, D. In-Bed Human Pose Estimation from Unseen and Privacy-Preserving Image Domains. In2022 IEEE 19th International Symposium on Biomedical Imaging (ISBI) 2022, 1-5 (2022).
59 Chang, Q. et al. Synthetic learning: Learn from distributed asynchronized discriminator GaN without sharing medical image data. in Proceedings of the IEEE Computer Society Conference on Computer Vision and Pattern Recognition, 13853-13863 (2020).
60 Charachon, M., Cournède, P. H., Hudelot, C. & Ardon, R. Leveraging conditional generative models in a general explanation framework of classifier decisions. Future Generation Computer Systems 132, 223-238 (2022). https://doi.org:10.1016/j.future.2022.02.020
61 Chen, D., Yu, N., Zhang, Y. & Fritz, M. GAN-Leaks: A Taxonomy of Membership Inference Attacks against Generative Models. in Proceedings of the ACM Conference on Computer and Communications Security 2020, 343-362 (2020).
62 Chen, J. et al. Stroke Risk Prediction with Hybrid Deep Transfer Learning Framework. IEEE Journal of Biomedical and Health Informatics 26, 411-422 (2022). https://doi.org:10.1109/JBHI.2021.3088750
63 Chen, J., Mowlaei, M. E. & Shi, X. Population-scale Genomic Data Augmentation Based on Conditional Generative Adversarial Networks. in Proceedings of the 11th ACM International Conference on Bioinformatics, Computational Biology and Health Informatics, BCB 2020, 1-6 (2020).
64 Chen, Z., Cao, B., Edwards, A., Deng, H. W. & Zhang, K. A deep imputation and inference framework for estimating personalized and race-specific causal effects of genomic alterations on PSA. Journal of Bioinformatics and Computational Biology 19, 2150016 (2021). https://doi.org:10.1142/S0219720021500165
65 Coyner, A. S. et al. Synthetic Medical Images for Robust, Privacy-Preserving Training of Artificial Intelligence: Application to Retinopathy of Prematurity Diagnosis. Ophthalmol Sci 2, 100126 (2022). https://doi.org:10.1016/j.xops.2022.100126
66 Devi, Y. S. & Kumar, S. P. Diabetic Retinopathy (DR) Image Synthesis Using DCGAN and Classification of DR Using Transfer Learning Approaches. International Journal of Image and Graphics 2023, 2340009 (2023). https://doi.org:10.1142/S0219467823400090
67 Dhami, D. S., Das, M. & Natarajan, S. Beyond Simple Images: Human Knowledge-Guided GANs for Clinical Data Generation. in Proceedings of the 18th International Conference on Principles of Knowledge Representation and Reasoning, KR 2021, 247-257 (2021).
68 Diamantis, D. E., Gatoula, P. & Iakovidis, D. K. EndoVAE: Generating Endoscopic Images with a Variational Autoencoder. in IVMSP 2022 - 2022 IEEE 14th Image, Video, and Multidimensional Signal Processing Workshop, 1-5 (2022).
69 Diller, G. P. et al. Utility of deep learning networks for the generation of artificial cardiac magnetic resonance images in congenital heart disease. BMC Medical Imaging 20, 1-8 (2020). https://doi.org:10.1186/s12880-020-00511-1

70 Ding, Y. et al. DeepKeyGen: A Deep Learning-Based Stream Cipher Generator for Medical Image Encryption and Decryption. IEEE Trans Neural Netw Learn Syst 33, 4915-4929 (2022). https://doi.org:10.1109/tnnls.2021.3062754
71 Ding, Y. et al. DeepEDN: A Deep-Learning-Based Image Encryption and Decryption Network for Internet of Medical Things. IEEE Internet of Things Journal 8, 1504-1518 (2021). https://doi.org:10.1109/JIOT.2020.3012452
72 Sousa, M. Q. et al. Sousa MQ, Pedrosa J, Rocha J, Pereira SC, Mendonça AM, Campilho A. Chest Radiography Few-Shot Image Synthesis for Automated Pathology Screening Applications. In2021 IEEE International Conference on Bioinformatics and Biomedicine (BIBM), 1791-1798 (2021).
73 Finck, T. et al. Uncertainty-Aware and Lesion-Specific Image Synthesis in Multiple Sclerosis Magnetic Resonance Imaging: A Multicentric Validation Study. Frontiers in Neuroscience 16, 889808 (2022). https://doi.org:10.3389/fnins.2022.889808
74 Foomani, F. H. et al. Synthesizing time-series wound prognosis factors from electronic medical records using generative adversarial networks. Journal of Biomedical Informatics 125, 103972 (2022). https://doi.org:10.1016/j.jbi.2021.103972
75 Gao, H. & Ogawara, K. Gao H, Ogawara K. Adaptive data generation and bidirectional mapping for polyp images. In2020 IEEE Applied Imagery Pattern Recognition Workshop (AIPR), 1-6 (2020).
76 Garrucho, L. et al. High-resolution synthesis of high-density breast mammograms: Application to improved fairness in deep learning based mass detection. Front Oncol 12, 1044496 (2022). https://doi.org:10.3389/fonc.2022.1044496
77 Ghosh, S., Boucher, C., Bian, J. & Prosperi, M. Propensity score synthetic augmentation matching using generative adversarial networks (PSSAM-GAN). Comput Methods Programs Biomed Update 1, 100020 (2021). https://doi.org:10.1016/j.cmpbup.2021.100020
78 Ghosh, S., Feng, Z., Bian, J., Butler, K. & Prosperi, M. DR-VIDAL - Doubly Robust Variational Information-theoretic Deep Adversarial Learning for Counterfactual Prediction and Treatment Effect Estimation on Real World Data. AMIA Annu Symp Proc, 485-494 (2022).
79 Gu, Y., Peng, Y. & Li, H. AIDS Brain MRIs Synthesis via Generative Adversarial Networks Based on Attention-Encoder. in 2020 IEEE 6th International Conference on Computer and Communications, ICCC, 629-633 (2020).
80 Guan, J. Q., Li, R. Z., Yu, S. & Zhang, X. G. A Method for Generating Synthetic Electronic Medical Record Text. IEEE-ACM Transactions on Computational Biology and Bioinformatics 18, 173-182 (2021). https://doi.org:10.1109/TCBB.2019.2948985
81 Han, G. et al. The Detection and Segmentation of Pulmonary Nodules Based on U-Net. in Lecture Notes in Computer Science (including subseries Lecture Notes in Artificial Intelligence and Lecture Notes in Bioinformatics), 236-244 (2021).
82 Haoqi, G. & Ogawara, K. CGAN-based synthetic medical image augmentation between retinal fundus images and vessel segmented images. in 2020 5th International Conference on Control and Robotics Engineering, ICCRE, 218-223 (2020).
83 He, Y., Fu, B., Yu, J., Li, R. F. & Jiang, R. C. Efficient Learning of Healthcare Data from IoT Devices by Edge Convolution Neural Networks. Applied Sciences-Basel 10, 1-19 (2020). https://doi.org:10.3390/app10248934
84 Hu, R., Li, D., Ng, S. K. & Zheng, Z. CB-GAN: Generate Sensitive Data with a Convolutional Bidirectional Generative Adversarial Networks. in Lecture Notes in Computer Science (including subseries Lecture Notes in Artificial Intelligence and Lecture Notes in Bioinformatics), 159-174 (2023).

85 Hussain, F., Tbarki, K. & Ksantini, R. GAN-based one-class classification SVM for real time medical image intrusion detection. International Journal of Computing and Digital Systems 13, 625-641 (2023). https://doi.org:10.12785/ijcds/130150
86 Imtiaz, S., Arsalan, M., Vlassov, V. & Sadre, R. Synthetic and Private Smart Health Care Data Generation using GANs. In 2021 International Conference on Computer Communications and Networks (ICCCN), 1-7 (2021).
87 Iqball, T. & Wani, M. A. X-ray images dataset augmentation with progressively growing generative adversarial network. in Proceedings of the 2021 8th International Conference on Computing for Sustainable Global Development, INDIACom 2021, 93-97 (2021).
88 Kugelman, J., Alonso-Caneiro, D., Read, S. A., Vincent, S. J. & Collins, M. J. Semi-supervised learning with cross-localisation in shared GAN latent space for enhanced OCT data augmentation. in 2022 International Conference on Digital Image Computing: Techniques and Applications, DICTA 2022, 1-7 (2022).
89 Jeon, M., Park, H., Kim, H. J., Morley, M. & Cho, H. k-SALSA: k-Anonymous Synthetic Averaging of Retinal Images via Local Style Alignment. in Lecture Notes in Computer Science (including subseries Lecture Notes in Artificial Intelligence and Lecture Notes in Bioinformatics), 661-678 (2022).
90 Kamli, A., Saouli, R., Batatia, H., Ben Naceur, M. B. & Youkana, I. Synthetic medical image generator for data augmentation and anonymisation based on generative adversarial network for glioblastoma tumors growth prediction. IET Image Processing 14, 4248-4257 (2020). https://doi.org:10.1049/iet-ipr.2020.1141
91 Kaur, S., Kumar, S. & Homayouni, H. Synthetic High-Resolution COVID-19 Chest X-Ray Generation. In Proceedings of the 2023 Australasian Computer Science Week, 151-159 (2023).
92 Kazemi, A., Li, Z., Peréz-Rosas, V. & Mihalcea, R. Extractive and Abstractive Explanations for Fact-Checking and Evaluation of News. in NLP4IF 2021 - NLP for Internet Freedom: Censorship, Disinformation, and Propaganda, Proceedings of the 4th Workshop, 45-50 (2021).
93 Khader, F. et al. Denoising diffusion probabilistic models for 3D medical image generation. Scientific Reports 13, 7303 (2023). https://doi.org:10.1038/s41598-023-34341-2
94 Khosravi, B. et al. Creating High Fidelity Synthetic Pelvis Radiographs Using Generative Adversarial Networks: Unlocking the Potential of Deep Learning Models Without Patient Privacy Concerns. J Arthroplasty 38, 2037-2043 (2022). https://doi.org:10.1016/j.arth.2022.12.013
95 Kim, Y., Lee, J. H., Kim, C., Jin, K. N. & Park, C. M. GAN based ROI conditioned synthesis of medical image for data augmentation. In Medical Imaging 2023: Image Processing, 739-745 (2023).
96 Krishna, A. A., Arikutharam, V., Ramnan, K. V., Bharathi, H. & Chandar, T. S. Dynamic Image Encryption using Neural Networks for Medical Images. in 2022 IEEE IAS Global Conference on Emerging Technologies, GlobConET, 739-745 (2022).
97 Kumah-Crystal, Y., Mankowitz, S., Embi, P. & Lehmann, C. U. ChatGPT and the clinical informatics board examination: the end of unproctored maintenance of certification?. Journal of the American Medical Informatics Association 19, ocad104. (2023).
98 Kumar, R. et al. Permissioned Blockchain and Deep Learning for Secure and Efficient Data Sharing in Industrial Healthcare Systems. IEEE Transactions on Industrial Informatics 18, 8065-8073 (2022). https://doi.org:10.1109/TII.2022.3161631

99 Kuo, N. I. H. et al. Generating synthetic clinical data that capture class imbalanced distributions with generative adversarial networks: Example using antiretroviral therapy for HIV. Journal of biomedical informatics, 104436 (2023). https://doi.org:10.1016/j.jbi.2023.104436
100 Lee, D. et al. Generating sequential electronic health records using dual adversarial autoencoder. Journal of the American Medical Informatics Association 27, 1411-1419 (2020). https://doi.org:10.1093/jamia/ocaa119
101 Li, J. F. et al. Are synthetic clinical notes useful for real natural language processing tasks: A case study on clinical entity recognition. Journal of the American Medical Informatics Association 28, 2193-2201 (2021). https://doi.org:10.1093/jamia/ocab112
102 Li, J., Cairns, B. J., Li, J. S. & Zhu, T. T. Generating synthetic mixed-type longitudinal electronic health records for artificial intelligent applications. NPJ Digital Medicine 6, 98 (2023). https://doi.org:10.1038/s41746-023-00834-7
103 Li, J. et al. A multicenter random forest model for effective prognosis prediction in collaborative clinical research network. Artificial Intelligence in Medicine 103, 101814 (2020). https://doi.org:10.1016/j.artmed.2020.101814
104 Li, R. et al. Improving an Electronic Health Record-Based Clinical Prediction Model Under Label Deficiency: Network-Based Generative Adversarial Semisupervised Approach. JMIR Med Inform 11, e47862 (2023). https://doi.org:10.2196/47862
105 Li, Z. et al. ChatDoctor: A Medical Chat Model Fine-Tuned on a Large Language Model Meta-AI (LLaMA) Using Medical Domain Knowledge. Cureus 15 (2023). https://doi.org:10.7759/cureus.40895
106 Liu, J. N. et al. Application of QR Code Watermarking and Encryption in the Protection of Data Privacy of Intelligent Mouth-Opening Trainer. IEEE Internet of Things Journal 10, 10510-10518 (2023). https://doi.org:10.1109/JIOT.2023.3242319
107 Liu, S. R. et al. Using AI-generated suggestions from ChatGPT to optimize clinical decision support. Journal of the American Medical Informatics Association 30, 1237-1245 (2023). https://doi.org:10.1093/jamia/ocad072
108 Liu, W. K. et al. BFG: privacy protection framework for internet of medical things based on blockchain and federated learning. Connection Science 35, 2199951 (2023). https://doi.org:10.1080/09540091.2023.2199951
109 Liu, Y., Peng, J., Yu, J. J. Q. & Wu, Y. Ppgan: Privacy-preserving generative adversarial network. in Proceedings of the International Conference on Parallel and Distributed Systems – ICPADS, 985-989 (2019).
110 Liu, Z., Li, X. & Yu, P. Mitigating health disparities in EHR via deconfounder. in Proceedings of the 13th ACM International Conference on Bioinformatics, Computational Biology and Health Informatics, BCB, 1-6 (2022).
111 Mahmood, F., Chen, R. & Durr, N. J. Unsupervised Reverse Domain Adaptation for Synthetic Medical Images via Adversarial Training. IEEE Transactions on Medical Imaging 37, 2572-2581 (2018). https://doi.org:10.1109/TMI.2018.2842767
112 Mikołajczyk, A., Majchrowska, S. & Carrasco Limeros, S. The (de)biasing Effect of GAN-Based Augmentation Methods on Skin Lesion Images. in Lecture Notes in Computer Science (including subseries Lecture Notes in Artificial Intelligence and Lecture Notes in Bioinformatics), 437-447 (2022).
113 Moghadam, P. A. et al. A Morphology Focused Diffusion Probabilistic Model for Synthesis of Histopathology Images. in Proceedings - 2023 IEEE Winter Conference on Applications of Computer Vision, WACV 2023, 1999-2008 (2023).

114 Mohanty, A., Sutherland, A., Bezbradica, M. & Javidnia, H. Towards Synthetic Generation of Clinical Rosacea Images with GAN Models. in 2022 33rd Irish Signals and Systems Conference, ISSC, 1-5 (2022).
115 Montenegro, H., Silva, W. & Cardoso, J. S. Disentangled Representation Learning for Privacy-Preserving Case-Based Explanations. in Lecture Notes in Computer Science (including subseries Lecture Notes in Artificial Intelligence and Lecture Notes in Bioinformatics), 33-45 (2023).
116 Moreu, E., McGuinness, K. & O'Connor, N. E. Synthetic Data for Unsupervised Polyp Segmentation. in CEUR Workshop Proceedings, 96-105 (2021).
117 Nasution, B. I., Bhaswara, I. D., Nugraha, Y. & Kanggrawan, J. I. Data Analysis and Synthesis of COVID-19 Patients using Deep Generative Models: A Case Study of Jakarta, Indonesia. in ISC2 2022 - 8th IEEE International Smart Cities Conference, 1-7 (2022).
118 Ng, M. F. & Hargreaves, C. A. Generative Adversarial Networks for the Synthesis of Chest X-ray Images. Engineering Proceedings 31, 84 (2023). https://doi.org:10.3390/ASEC2022-13954
119 Nguyen, L. X., Aung, P. S., Le, H. Q., Park, S. B. & Hong, C. S. A New Chapter for Medical Image Generation: The Stable Diffusion Method. in International Conference on Information Networking, 483-486 (2023).
120 Nik, A. H. Z., Riegler, M. A., Halvorsen, P. & Storås, A. M. Generation of Synthetic Tabular Healthcare Data Using Generative Adversarial Networks. Generation of Synthetic Tabular Healthcare Data Using Generative Adversarial Networks. in Lecture Notes in Computer Science (including subseries Lecture Notes in Artificial Intelligence and Lecture Notes in Bioinformatics), 434-446 (2023).
121 Nyamathulla, S., Meghana, C. S. & Yasaswi, K. Brain Tumour Segmentation Using Wasserstein Generative Adversarial Networks(WGANs). in 7th International Conference on Trends in Electronics and Informatics, ICOEI 2023 – Proceedings, 1571-1577 (2023).
122 Ong, H. et al. GPT Technology to Help Address Longstanding Barriers to Care in Free Medical Clinics. Annals of Biomedical Engineering 51, 1906-1909 (2023). https://doi.org:10.1007/s10439-023-03256-4
123 Ozyigit, E. B., Arvanitis, T. N. & Despotou, G. Generation of Realistic Synthetic Validation Healthcare Datasets Using Generative Adversarial Networks. Importance of Health Informatics in Public Health During A Pandemic 272, 322-325 (2020).
124 Pan, X. D., Zhang, M., Ji, S. L. & Yang, M. Privacy risks of general-purpose language models. in 2020 IEEE Symposium on Security and Privacy (SP 2020), 1314-1331 (2020).
125 Pandey, A., Paliwal, B., Dhall, A., Subramanian, R. & Mahapatra, D. This Explains That: Congruent Image-Report Generation for Explainable Medical Image Analysis with Cyclic Generative Adversarial Networks. Interpretability of Machine Intelligence in Medical Image Computing, and Topological Data Analysis and Its Applications for Medical Data 12929, 34-43 (2021).
126 Pastorino, J. & Biswas, A. K. Data adequacy bias impact in a data-blinded semi-supervised GAN for privacy-Aware COVID-19 chest X-ray classification. in Proceedings of the 13th ACM International Conference on Bioinformatics, Computational Biology and Health Informatics, BCB, 1-8 (2022).

127 Pattanayak, S. & Ludwig, S. A. Analyzing Privacy of Time Series Data Using Substitute Auto-encoder Neural Network. in 2020 IEEE Symposium Series on Computational Intelligence, SSCI 2020, 1411-1418 (2020).
128 Paul, W., Cao, Y., Zhang, M. & Burlina, P. Defending Medical Image Diagnostics Against Privacy Attacks Using Generative Methods: Application to Retinal Diagnostics. in Lecture Notes in Computer Science (including subseries Lecture Notes in Artificial Intelligence and Lecture Notes in Bioinformatics), 174-187 (2021).
129 Rajotte, J. F. et al. Reducing bias and increasing utility by federated generative modeling of medical images using a centralized adversary. in GoodIT 2021 - Proceedings of the 2021 Conference on Information Technology for Social Good, 79-84 (2021).
130 Rashidian, S. et al. SMOOTH-GAN: Towards Sharp and Smooth Synthetic EHR Data Generation. in Artificial Intelligence in Medicine (AIME 2020), 37-48 (2020).
131 Rayavarapu, S. M., Prashanthi, T. S., Kumar, G. S., Lavanya, Y. L. & Rao, G. S. A Generative Adversarial Network Based Approach for Synthesis of Deep Fake Electrocardiograms. International Journal on Recent and Innovation Trends in Computing and Communication 11, 223-227 (2023). https://doi.org:10.17762/ijritcc.v11i3.6340
132 Rejusha, R. R. T. & Vipin Kumar, S. V. K. Artificial MRI Image Generation using Deep Convolutional GAN and its Comparison with other Augmentation Methods. in ICCISc 2021 - 2021 International Conference on Communication, Control and Information Sciences, Proceedings Volume 1, 1-6 (2021).
133 Ren, H., Wang, J. & Zhao, W. X. Generative Adversarial Networks Enhanced Pre-training for Insufficient Electronic Health Records Modeling. in Proceedings of the ACM SIGKDD International Conference on Knowledge Discovery and Data Mining, 3810-3818 (2022).
134 Rodriguez-Almeida, A. J. et al. Synthetic Patient Data Generation and Evaluation in Disease Prediction Using Small and Imbalanced Datasets. IEEE Journal of Biomedical and Health Informatics 27, 2670-2680 (2023). https://doi.org:10.1109/JBHI.2022.3196697
135 Sakai, T., Seo, M., Matsushiro, N. & Chen, Y. W. Simulation of Facial Palsy using Conditional Generative Adversarial Networks and Face Shape Normalization. in 2021 IEEE 10th Global Conference on Consumer Electronics, GCCE 2021, 793-797 (2021).
136 Sakai, T., Seo, M., Matsushiro, N. & Chen, Y. W. Simulation of Facial Palsy Using Cycle GAN with Skip-Layer Excitation Module and Self-Supervised Discriminator. Journal of Image and Graphics(United Kingdom) 11, 132-139 (2023). https://doi.org:10.18178/joig.11.2.132-139
137 Sakai, T., Seo, M., Matushiro, N. & Chen, Y. W. Simulation of Facial Palsy using an Improved Cycle GAN and Face Restoration Network. in 2023 IEEE International Conference on Consumer Electronics, ICCE, 1-4 (2023).
138 Schutte, A. D. et al. Overcoming barriers to data sharing with medical image generation: a comprehensive evaluation. NPJ Digital Medicine 4, 141 (2021). https://doi.org:10.1038/s41746-021-00507-3
139 Selvaganapathy, S. G. & Sadasivam, S. Healthcare Security: Usage of Generative Models for Malware Adversarial Attacks and Defense. in Lecture Notes in Networks and Systems, 885-897 (2021).
140 Sengupta, S., Athwale, A., Gulati, T., Zelek, J. & Lakshminarayanan, V. Funsyn-Net: Enhanced residual variational auto-encoder and image-to-image translation network for fundus image synthesis. in MEDICAL IMAGING 2020: Image Processing 11313, 665-671 (2021).

141 Sharafudeen, M., Andrew, J. & Chandra, V. S. S. Leveraging Vision Attention Transformers for Detection of Artificially Synthesized Dermoscopic Lesion Deepfakes Using Derm-CGAN. DIAGNOSTICS 13, 825 (2023). https://doi.org:10.3390/diagnostics13050825
142 Sharafudeen, M. & Chandra, S. S. V. Medical Deepfake Detection using 3-Dimensional Neural Learning. in Artificial Neural Networks in Pattern Recognition, ANNPR 2022 Vol.13739, 169-180 (2023).
143 Shen, Z. et al. DeformableGAN: Generating Medical Images With Improved Integrity for Healthcare Cyber Physical Systems. IEEE Transactions on Network Science and Engineering, 1-13 (2022). https://doi.org:10.1109/TNSE.2022.3190765
144 Shi, J. P., Wang, D., Tesei, G. & Norgeot, B. Generating high-fidelity privacy-conscious synthetic patient data for causal effect estimation with multiple treatments. Frontiers in Artificial Intelligence 5, 918813 (2022). https://doi.org:10.3389/frai.2022.918813
145 Shin, K. et al. An Image Turing Test on Realistic Gastroscopy Images Generated by Using the Progressive Growing of Generative Adversarial Networks. Journal of Digital Imaging 36, 1760-1769 (2023). https://doi.org:10.1007/s10278-023-00803-2
146 Sindhura, D., Pai, R. M., Bhat, S. N. & Pai, M. M. Sub-Axial Vertebral Column Fracture CT Image Synthesis by Progressive Growing Generative Adversarial Networks (PGGANs). in 2022 IEEE International Conference on Distributed Computing, VLSI, Electrical Circuits and Robotics, DISCOVER 2022 – Proceedings, 311-315 (2022).
147 Sliman, H. et al. MedWGAN based synthetic dataset generation for Uveitis pathology. Intelligent Systems with Applications 18, 200223 (2023). https://doi.org:10.1016/j.iswa.2023.200223
148 Sorin, V. et al. Large language model (ChatGPT) as a support tool for breast tumor board. NPJ Breast Cancer 9, 44 (2023). https://doi.org:10.1038/s41523-023-00557-8
149 Sreedha, B., Nair, P. R. & Maity, R. Non-invasive early diagnosis of jaundice with computer vision. Procedia Computer Science 218, 1321-1334 (2023).
150 Sun, S. et al. Generating Longitudinal Synthetic EHR Data with Recurrent Autoencoders and Generative Adversarial Networks. in Lecture Notes in Computer Science (including subseries Lecture Notes in Artificial Intelligence and Lecture Notes in Bioinformatics), 153-165 (2021).
151 Sun, Y., Yuan, P. S. & Sun, Y. M. MM-GAN: 3D MRI data augmentation for medical image segmentation via generative adversarial networks. in 11TH IEEE International Conference on Knowledge Graph (ICKG 2020), 227-234 (2020).
152 Szafranowska, Z. et al. Sharing Generative Models Instead of Private Data: A Simulation Study on Mammography Patch Classification. in Proceedings of SPIE - The International Society for Optical Engineering 12286, 169-177. (2022)
153 Takahashi, Y. et al. Decentralized Learning with Virtual Patients for Medical Diagnosis of Diabetes. SN Computer Science 2, 1-10 (2021). https://doi.org:10.1007/s42979-021-00564-1
154 Tang, Z., Chai, X., Lu, Y., Wang, B. & Tan, Y. An end-to-end screen shooting resilient blind watermarking scheme for medical images. Journal of Information Security and Applications 76, 103547 (2023). https://doi.org:10.1016/j.jisa.2023.103547
155 Tariq, U. et al. Brain Tumor Synthetic Data Generation with Adaptive StyleGANs. in Communications in Computer and Information Science, 147-159 (2023).

156 Thambawita, V. et al. DeepSynthBody: The beginning of the end for data deficiency in medicine. in 2021 International Conference on Applied Artificial Intelligence, ICAPAI, 1-8 (2021).
157 Thambawita, V. et al. SinGAN-Seg: Synthetic training data generation for medical image segmentation. PLoS One 17, e0267976 (2022). https://doi.org:10.1371/journal.pone.0267976
158 Tian, Q. et al. ConfounderGAN: Protecting Image Data Privacy with Causal Confounder. in Advances in Neural Information Processing Systems 35, 32789-32800 (2022).
159 Torfi, A. & Fox, E. A. CorGAN: Correlation-capturing convolutional generative adversarial networks for generating synthetic healthcare records. in Proceedings of the 33rd International Florida Artificial Intelligence Research Society Conference, FLAIRS 2020, 335-340 (2020).
160 Torrents-Barrena, J. et al. Fetal MRI Synthesis via Balanced Auto-Encoder Based Generative Adversarial Networks. in 2018 40TH Annual International Conference Of The Ieee Engineering in Medicine And Biology Society (EMBC), 2599-2602 (2018).
161 Trinh, N. H. & O'Brien, D. Generative adversarial network-based semi-supervised learning for pathological speech classification. in Lecture Notes in Computer Science (including subseries Lecture Notes in Artificial Intelligence and Lecture Notes in Bioinformatics), 169-181 (2020).
162 Vatanparvar, K. et al. A generative model for speech segmentation and obfuscation for remote health monitoring. in 2019 IEEE 16TH International Conference on Wearable and Implantable Body Sensor Networks (BSN), 1-4 (2019).
163 Venu, S. K. Improving the Generalization of Deep Learning Classification Models in Medical Imaging Using Transfer Learning and Generative Adversarial Networks. in Agents and Artificial Intelligence, ICAART 2021 Vol. 13251, 218-235 (2022).
164 Wang, C. et al. Toward MR-only proton therapy planning for pediatric brain tumors: Synthesis of relative proton stopping power images with multiple sequence MRI and development of an online quality assurance tool. Medical Physics 49, 1559-1570 (2022). https://doi.org:10.1002/mp.15479
165 Wang, J. B. et al. FedMed-GAN: Federated domain translation on unsupervised cross- modality brain image synthesis. Neurocomputing 546, 126282 (2023). https://doi.org:10.1016/j.neucom.2023.126282
166 Wang, J. et al. FedMed-ATL: Misaligned Unpaired Cross-Modality Neuroimage Synthesis via Affine Transform Loss. in MM 2022 - Proceedings of the 30th ACM International Conference on Multimedia, 1522-1531 (2022).
167 Wang, L., Zhang, W. & He, X. F. Continuous patient-centric sequence generation via sequentially coupled adversarial learning. in Database Systems for Advanced Applications (DASFAA 2019), PT II Vol. 11447, 36-52 (2019).
168 Wang, S., Rudolph, C., Nepal, S., Grobler, M. & Chen, S. Y. PART-GAN: Privacy-Preserving Time-Series Sharing. in Artificial Neural Networks and Machine Learning, ICANN 2020, PT I Vol. 12396, 578-593 (2020).
169 Wang, S. Q. et al. Diabetic Retinopathy Diagnosis Using Multichannel Generative Adversarial Network With Semisupervision. IEEE Transactions on Automation Science and Engineering 18, 574-585 (2021). https://doi.org:10.1109/TASE.2020.2981637

170 Wang, X. et al. Federated Learning-Empowered Disease Diagnosis Mechanism in the Internet of Medical Things: From the Privacy-Preservation Perspective. IEEE Transactions on Industrial Informatics 19, 7905-7913 (2023). https://doi.org:10.1109/TII.2022.3210597
171 Wang, Y. et al. Human Gait Data Augmentation and Trajectory Prediction for Lower-Limb Rehabilitation Robot Control Using GANs and Attention Mechanism. Machines 9, 367 (2021). https://doi.org:10.3390/machines9120367
172 Wang, Y. Y., Meng, X. J. & Liu, X. M. Differentially Private Recurrent Variational Autoencoder For Text Privacy Preservation. Mobile Networks and Applications, 1-16 (2023). https://doi.org:10.1007/s11036-023-02096-9
173 Wang, Z. R. et al. Synthetic artificial intelligence using generative adversarial network for retinal imaging in detection of age-related macular degeneration. Frontiers in Medicine 10 (2023). https://doi.org:10.3389/fmed.2023.1184892
174 Wang, Z. et al. Generative Data Augmentation for Non-IID Problem in Decentralized Clinical Machine Learning. in Proceedings - 2022 4th International Conference on Data Intelligence and Security, ICDIS 2022, 336-343 (2022).
175 Wang, Z. et al. Generation of synthetic ground glass opacities (GGOs) using generative adversarial networks (GANs). Annals of Oncology 33, S80 (2022). https://doi.org:10.1016/j.annonc.2022.02.132
176 Wu, C. L. et al. Vessel-GAN: Angiographic reconstructions from myocardial CT perfusion with explainable generative adversarial networks. Future Generation Computer Systems-The International Journal of EScience 130, 128-139 (2022). https://doi.org:10.1016/j.future.2021.12.007
177 Xiang, X. et al. From One-hot Encoding to Privacy-preserving Synthetic Electronic Health Records Embedding. in ACM International Conference Proceeding Series, 407-413 (2020).
178 Xu, Q. Y., Ahmadi, E., Amini, A., Rus, D. & Lo, A. W. Identifying and Mitigating Potential Biases in Predicting Drug Approvals. Drug Safety 45, 521-533 (2022). https://doi.org:10.1007/s40264-022-01160-9
179 Xue, S. et al. A cross-scanner and cross-tracer deep learning method for the recovery of standard-dose imaging quality from low-dose PET. European Journal of Nuclear Medicine and Molecular Imaging 49, 1843-1856 (2022). https://doi.org:10.1007/s00259-021-05644-1
180 Yale, A. et al. Assessing privacy and quality of synthetic health data. In Proceedings of the Conference on Artificial Intelligence for Data Discovery and Reuse, 1-4 (2019).
181 Yale, A. et al. Privacy preserving synthetic health data. in ESANN 2019 - Proceedings, 27th European Symposium on Artificial Neural Networks, Computational Intelligence and Machine Learning, 465-470 (2019).
182 Yan, Z. Q., Wicaksana, J., Wang, Z. W., Yang, X. & Cheng, K. T. Variation-Aware Federated Learning With Multi-Source Decentralized Medical Image Data. IEEE Journal of Biomedical and Health Informatics 25, 2615-2628 (2021). https://doi.org:10.1109/JBHI.2020.3040015
183 Yang, H., Ge, M., Xiang, K., Bai, X. & Li, H. FedVAE: Communication-Efficient Federated Learning With Non-IID Private Data. IEEE Systems Journal, 1-11 (2023). https://doi.org:10.1109/JSYST.2023.3274197
184 Yao, L., Zhang, Y., Zheng, Z. & Wu, G. GAN-based Differential Privacy Trajectory Data Publishing with Sensitive Label. in Proceedings - 2022 8th International Conference on Big Data Computing and Communications, BigCom 2022, 112-119 (2022).

185 Zhang, J. N., Zhu, E., Guo, X. F., Chen, H. H. & Yin, J. P. in Theoretical Computer Science (NCTCS 2018) Vol. 882, 150-158 (2018).
186 Zhang, L. L. et al. FedDPGAN: Federated Differentially Private Generative Adversarial Networks Framework for the Detection of COVID-19 Pneumonia. Information Systems Frontiers 23, 1403-1415 (2021). https://doi.org:10.1007/s10796-021-10144-6
187 Zhang, Z. Q., Yan, C., Lasko, T. A., Sun, J. M. & Malin, B. A. SynTEG: a framework for temporal structured electronic health data simulation. Journal of the American Medical Informatics Association 28, 596-604 (2021). https://doi.org:10.1093/jamia/ocaa262
188 Zhang, Z. Q., Yan, C. & Malin, B. A. Keeping synthetic patients on track: feedback mechanisms to mitigate performance drift in longitudinal health data simulation. Journal of the American Medical Informatics Association 29, 1890-1898 (2022). https://doi.org:10.1093/jamia/ocac131
189 Zhang, Z. Q., Yan, C., Mesa, D. A., Sun, J. M. & Malin, B. A. Ensuring electronic medical record simulation through better training, modeling, and evaluation. Journal of the American Medical Informatics Association 27, 99-108 (2020). https://doi.org:10.1093/jamia/ocz161
190 Zhao, L. Y. & Huang, J. J. A distribution information sharing federated learning approach for medical image data. Complex & Intelligent Systems 9, 5625-5636 (2023). https://doi.org:10.1007/s40747-023-01035-1
191 Zhou, C., Zhang, W., Chen, H. & Chen, L. Domain Adaptation for Medical Image Classification without Source Data. in Proceedings - 2022 IEEE International Conference on Bioinformatics and Biomedicine, BIBM 2022, 2224-2230 (2022).
192 Zhu, F., Ye, F., Fu, Y. C., Liu, Q. & Shen, B. R. Electrocardiogram generation with a bidirectional LSTM-CNN generative adversarial network. Scientific Reports 9, 6734 (2019). https://doi.org:10.1038/s41598-019-42516-z
193 el Hassouni, A., Hoogendoorn, M. & Muhonen, V. Using generative adversarial networks to develop a realistic human behavior simulator. in Lecture Notes in Computer Science (including subseries Lecture Notes in Artificial Intelligence and Lecture Notes in Bioinformatics), 476-483 (2018).
